# Genetic sequencing of the airborne fungal spectrum and air quality at a public hospital in Mexico City

**DOI:** 10.1371/journal.pgph.0004784

**Published:** 2025-06-24

**Authors:** María Carmen Calderón-Ezquerro, Carolina Brunner-Mendoza, César Guerrero-Guerra, Alejandro Sanchez-Flores A., Ilse Salinas-Peralta, Conchita Toriello, Alfredo Ponce-de León, Carmen Isela Ortega-Rosas. C.I.

**Affiliations:** 1 Departamento de Ciencias Ambientales, Instituto de Ciencias de la Atmósfera y Cambio Climático, UNAM, Mexico City, México; 2 Departamento de Microbiología y Parasitología, Facultad de Medicina, UNAM, Mexico City, México; 3 Unidad Universitaria de Secuenciación Masiva y Bioinformática, Instituto de Biotecnología, UNAM, Morelos, México; 4 Instituto Nacional de Ciencias Médicas y Nutrición Salvador Zubiran, Mexico City, México; 5 Unidad Académica Hermosillo, Universidad Estatal de Sonora, Sonora, México; Sri Ramachandra Institute of Higher Education and Research, INDIA

## Abstract

Hospital bioaerosols represent significant risks for nosocomial infections, contributing to patient morbidity and mortality. Exposure to these particles, particularly airborne fungal spores or propagules, can trigger adverse effects on the immune system and cause respiratory diseases. This study evaluated the airborne fungal community in a public hospital in Mexico City using a metagenomic approach, two types of aerobiological samplers as well as temperature, humidity, and suspended particle analysis. Sampling was carried out in three areas within the hospital: and outside the hospital. Airborne sampling was performed for three consecutive days, except in the EU. The results showed that using two different samplers revealed fungal diversity and composition variations. Specifically, the Cμ-Sampler captured a higher abundance and diversity of fungi than the AVPS, with Total Taxonomy Annotations at a Genus level of 626 in F1, 632 in F2, 485 in EU and 617 in OH). In the analysis of fungal presence, Ascomycota and Basidiomycota were identified as dominant phyla. Using the AVPS sampler, Ascomycota showed an overwhelming presence of 90% to 100% inside and outside the hospital, while Basidiomycota was found in a range of 1% to 10%. Using the CμS-Sampler, Ascomycota was observed to vary between 39% and 72% in areas F1 and F2 of the hospital and from 73% to 82% outside it. On the other hand, Basidiomycota presented values between 54% and 61% in F1 and from 18% to 27% outside the hospital. The predominant genera were *Aspergillus, Penicillium, Cladosporium* and *Alternaria*. The identification of twenty-seven fungal species, including opportunistic pathogens such as *Aspergillus fumigatus, Penicillium chrysogenum, P. expansum, Cladosporium* and *Alternaria alternata,* is a significant result of this study. The results revealed the diversity of fungi in the hospital environment. The proposed complementary use of different samplers could significantly optimise current surveillance methods.

## 1. Introduction

Bioaerosols in hospital environments have been identified as risk factors for various hospital-acquired infections (HAIs), which represent a significant cause of morbidity and mortality in hospitalized patients [1,2]. These bioaerosols, composed of airborne particles of biological origin, include bacteria, viruses, fungi, spores, cell fragments, and other organic compounds [3]. The health risk posed by these particles or bioaerosols is often related to their size and the type of microorganisms they carry [4,5]. Airborne particles, which range in size from 0.001 nm to 100 µm, have different compositions. Particles smaller than 5 µm can reach the lower respiratory tract, causing problems such as asthma, while those larger than 10 µm tend to settle in the upper respiratory tract.

It is estimated that a human inhales between 8,640 and 11,520 liters of air daily, which underlines the importance of assessing the quantity and diversity of respirable bioaerosols in the environment [6,7]. This knowledge is essential due to the direct impact that bioaerosols can have on human health. They can trigger allergies and cause infectious and non-infectious diseases, as well as acute and chronic toxic effects, including cancer, especially in susceptible individuals [8].

Exposure to these particles, especially fungal spores or propagules, can trigger adverse effects on the immune system and lead to respiratory diseases, such as asthma and allergies [9,10]. Activity within hospitals and the flow of people from outside also contribute to bioaerosols in the air. The diversity and quantity of fungi in the hospital environment depend on factors such as temperature, humidity, ventilation and maintenance of buildings, as well as their age and potential sources of contamination [11]. Excessive humidity can encourage fungal growth inside and outside environments such as hospitals, potentially increasing the prevalence of symptoms such as irritation, allergy and infection [12].

Species such as *Aspergillus* and *Penicillium* have been identified to proliferate in ventilation systems and on internal surfaces in hospitals [13]. In addition, fungi such as *Candida, Pneumocystis jirovecii* and Mucorales are responsible for fungal outbreaks associated with healthcare. *Candida* species detected in these environments *include C. albicans, C. glabrata, C. krusei* and *C. tropicalis,* as well as others such as *Cryptococcus, Trichosporon, Fusarium, Rhizopus* and *Rhizomucor* [14–17].

The presence of pathogenic, allergenic and mycotoxin-producing fungi in hospital air underlines their role as a route of disease transmission. Studies such as that by [9] have identified numerous thermophilic and mesophilic fungal species related to respiratory problems, such as *Aspergillus* spp., *Fusarium* spp. and *Penicillium* spp., in addition to some Mucorales such as *Rhizopus* and *Mucor*, which represent a significant risk for immunosuppressed people [11,18–20].

Many of these fungi produce toxins, glucans, and microbial volatile organic compounds, reinforcing the need to prevent the spread of airborne pathogens in hospitals to control nosocomial infections [21,22]. Species such as *Aspergillus fumigatus, Cladosporium, Alternaria* spp., *Mucor, Botrytis, Fusarium, Curvularia, Phoma* and *Rhizopus* have been identified as highly allergenic in these environments [11,22]. It is essential to highlight that many of the fungal species present in hospitals and their pathogenic or allergenic nature can also be associated with airborne particles, which amplifies their potential health risk. The spores of these fungi can be dispersed individually or attached to aerosols or particles, which increases their ability to penetrate the respiratory system [23]. For example, *Aspergillus fumigatus* has been reported to be associated with particles of 2.5 and 10 µm [24], while other studies have identified fungal species such as *Cladosporium, Alternaria, Fusarium, Penicillium* and *Aspergillus* in particles suspended in urban air [25].

Recently, in Sonora, northwestern Mexico, a study of airborne fungal spores [26] detected a high concentration of PM 10, 2.5, and 1 μm attached to fungal spores, mainly *Cladosporium* and *Alternaria*. A significant finding of this research is that most of the particles attached to the spores were nanoparticles (smaller than 1 μm), which are known to have adverse effects on human health. These findings highlight the importance of not only identifying the species of fungi present, but also the composition of the airborne particles they are associated with, as this poses a significant risk to the health of hospitalized patients.

Genetic sequencing has become an indispensable tool for identifying and characterising microorganisms, including fungi. In this study, we adopted an integrated genetic sequencing approach, combining next-generation sequencing (NGS) with Sanger sequencing. NGS allowed us to obtain a global view of the airborne fungal community, accurately identifying genera [27]. However, Sanger sequencing remains essential for accurate identification at the species level. This technique, which offers the resolution needed to distinguish between closely related species, complements the capabilities of NGS [28]. The integration of both techniques allows us to accurately identify fungi present in hospital air, which is crucial for understanding microbial ecology and its impact on health, especially in hospital environments where certain fungi can represent a significant risk. This underlines the need for continuous monitoring of fungi in these environments and the proposal of effective measures for their control [29], which will allow for proactive measures to be taken to manage this problem. Therefore, this study aimed to determine the composition of the airborne fungal community in four areas of a public health hospital during peak hours, using a metagenomic approach with two types of aerobiological samplers. In addition, the conditions of temperature, humidity, and concentration of aerodynamic particles suspended in the air were assessed to better understand the context in which these bioaerosols can affect human health.

## 2. Material and methods

### Statement of ethics

This research followed the highest ethical standards to ensure integrity, transparency, and respect for scientific studies’ ethical principles

### 2.1. Study area

The study was conducted at a hospital within the Mexico City Health System, located in the southern part of Mexico City (19°17′17″N 99°09′23″W). This hospital is a four-story building constructed with a robust reinforced concrete and steel structure to ensure stability. The interior spaces are divided by plasterboard or masonry walls, and the ceilings feature false ceilings with acoustic panels to enhance the ambience. Corridors, ranging in width from 2 to 4 meters and height from 2.4 to 3 meters (varying in specialized areas such as operating rooms), facilitate circulation throughout the four floors, with lengths adapted to the overall size of the hospital. This hospital includes departments such as internal medicine, highly specialized surgery, endocrinology, gastroenterology, hematology-oncology, infectious disease, nephrology and mineral metabolism, geriatric rheumatology, and cardio-pneumology. Hospital access was granted by the hospital’s internal authority and that, according to local regulations, no additional permits were necessary. Medical and paramedical staff routinely move between clinical rooms, diagnostic areas, operating rooms, and administrative offices. Staff flow is highest during shift changes (morning, afternoon, and evening) and during peak clinical hours (12:00–15:00 p.m.). Patients typically move from entry points (e.g., reception or emergency department) to consultation rooms, diagnostic areas, and wards. Inpatients remain on their assigned floors unless they are undergoing diagnostic tests or procedures. Visitors and family members, who often act as patient attendants, move primarily between waiting rooms, patient rooms, and occasionally cafeterias or designated visitor spaces. This movement is usually regulated by visiting hours and institutional policies. Sampling was performed in the mornings (between 8:00 a.m. and 14:00 p.m.) when the hospital had the highest influx of activity from doctors, nurses, cleaning and kitchen staff, patients, and some family members.

**Fig 1:**
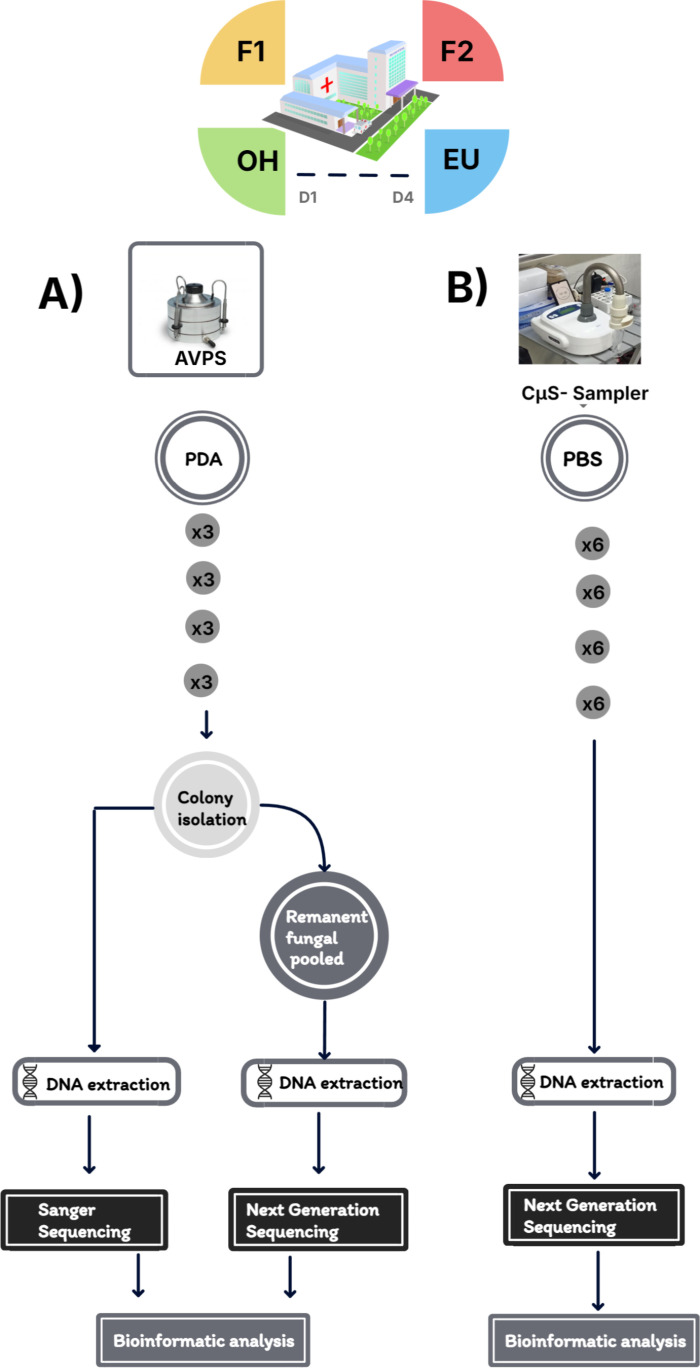
Methodological process for sampling airborne fungi inside and outside the hospital using the samplers A) AVPS and B) CμS-Sampler. A) shows the Potato dextrose agar culture medium used for the collection of airborne fungi, as well as the subsequent steps for their detection, either directly from the colonies developed and identified by Sanger Sequencing or from the pool of remaining fungi through their identification by Next Generation Sequencing (NGS). B) illustrates the use of PBS solution for collecting fungi from the air, DNA extraction, and identification of fungi by NGS. It also shows the number of replicates, sampling days, and monitored areas.

The sampling was carried out at the Emergency Unit (EU) near the nursing station, on Floor 1 (F1) and Floor 2 (F2) in the main hallway. On these floors are patients with various conditions; some are undergoing medical treatment, waiting for surgery, or in the postoperative stage. Sampling was also conducted outside the hospital (OH) at the main entrance of the hospital campus. Air sampling was performed over three consecutive days, except in the EU, where it was limited to two days due to hospital restrictions (June 14–16, 2022, and from June 21–23, 2022) (Fig 1). The hospital has 167 beds, of which F1 and F2 have 60 beds each, and the EU has 20 beds.

### 2.2. Aerobiological samplers

Airborne fungi were collected using an Andersen One-Stage Viable Particle Sampler (AVPS) (Thermo Fisher Scientific, USA) with a flow rate of 28.3 L/min. This sampler is an impaction-based air sampler that actively draws a measured volume of air through a single-stage impactor plate using a vacuum pump. Particles in the air, including fungi spores, are impacted onto a solid agar medium. This facilitates their subsequent identification by morphological features and by genetic sequencing [30,31]. Sampling was performed at a height of 1.5 meters for 15 minutes (with a total volume of 424.5 L/15 min), in triplicate in each hospital area and outside. The culture media used for sampling was Potato Dextrose Agar (PDA). Petri dishes were taken to the laboratory and incubated at 28 °C for 4–7 days (Fig 1A).

Airborne fungi were also collected with Coriolis μ sampler (CμS-Sampler) (Bertin Technologies, St-Berthely, France), which is a liquid-based air sampler that sucks in air at high flow rates through a cone-shaped air inlet. The airflow enters a cyclonic chamber, where centrifugal force separates airborne particles from the air. Instead of impacting onto a solid surface (like the AVPS), particles are trapped in a sterile liquid collection medium, in this study, 15 ml of phosphate-buffered saline solution (PBS) (Thermo Fisher Scientific) with a flow rate of 250 L/min. Sampling was performed at a height of 1.5 meters for 10 minutes (with a total volume of 2500 L/10 min), with six replicates in each hospital area and outside. All samples were transported in a cold chamber to the laboratory and stored at -20°C until molecular processing (Fig 1B).

### 2.3. Airborne particulate matter and environmental parameters

In each monitored area, the concentration of suspended particles with diameters of 0.5 μm, 1.0 μm, 2.5 μm, 5.0 μm, 10.0 μm and total particulate matter (TPM), temperature, and relative humidity were measured daily using a Model 8306 particle counter (Particles Plus). The airborne particulate matter and environmental parameters measurements were taken 1.5 meters above the ground. Sampling was conducted in cycles of 1 minute on, followed by 3 minutes off, during 2 hours. The total duration for particle measurement in each study area was approximately three hours, aligning with the airborne fungal sampling period.

### 2.4. Processing of the collected samples

In this study, our primary objective was to capture the widest diversity of airborne fungi. To achieve this, we utilized two types of samplers with distinct technologies and collection media (PDA and PBS). Our secondary challenge was to extract sufficient DNA from those air samples. We addressed this by isolating individual colonies and also recovering material from the remaining colonies post-isolation (Fig 1A).

We employed various molecular detection technologies to enhance our analysis. Next-generation sequencing (NGS) enabled us to identify a broader spectrum of fungi (Fig 1A and Fig 1B). In contrast, Sanger sequencing allowed for the identification of some fungal groups at the species level (Fig 1A).

#### 2.4.1. AVPS samples.

2.4.1.1. ***Process of samples obtained from fungi collected with the AVPS (***Fig 1A). Airborne fungi collected from the air in the potato dextrose agar (PDA) culture medium were individually isolated. Fungi were isolated by morphological identification. Isolates of fungi were maintained on PDA and stored in the dark at 4°C until the DNA extraction process (Fig 1A).

For Sanger sequencing the genomic DNA was extracted from fungal cultures by inoculating conidiospores from these isolates into 50 ml of 0.2% (w/v) yeast extract, 1% peptone, 2% glucose broth Yeast Extract Peptone Dextrose (YPD) in 125 ml flasks. The flasks were shaken at 200 rpm at 27 °C for 3–5 days. The mycelial mat was collected by vacuum filtration onto Fisherbrand P8 filter paper, washed with distilled water, and dried between two sterile paper filters. Approximately, 2 g of harvested mycelium was stored at -10°C. Genomic DNA was extracted using DNeasy Plant Mini Kit (Qiagen) according to the conditions described by the manufacturer. A DS-11 spectrophotometer (DeNovix) was used for quantification and purity of DNA. These results were also confirmed by gel electrophoresis using 0.8% agarose gel stained with SYBERSAFE. PCR amplification and sequencing of ITS rDNA region and Elongation Factor 1 alpha ITS rDNA region with the primers ITS1 (5´- TCCGTAGGTGAACCTGCGG-3’) and ITS4 (5´- ’TCCTCCGCTTATTGATATGC-3’) were used (Sigma-Aldrich, USA), as well as the EF1A region with the primers EF1-983F (5´-GCYCCYGGHCAYCGTGAYTTYAT) EF1-1567R (5´-ACHGTRCCRATACCACCRATCTT). PCR reactions were carried out in a total volume of 25 μL using the GoTaq Colorless Master Mix (Promega), following the manufacturer’s instructions. Amplifications were conducted on a BioRad T100 thermocycler, with thermal cycling conditions set according to the manufacturer’s recommended protocol. The amplification products were analyzed through 1.2% agarose gel electrophoresis with SYBR Safe in 0.5 X TBE buffer (45 mM Tris-Base, 45 mM boric acid, 1 mM EDTA). PCR products were sequenced by Sanger at PSOmagen, Inc., using the same primers described above.

2.4.1.2. ***Process of samples obtained from fungal mix collected with the AVPS*** (Fig 1B). For NGS sequencing, after isolation of fungal colonies, the Petri dishes with PDA medium were not discarded; 5 ml of PBS solution was added to them, and the surface of the culture medium of each dish was swept with an L-shaped inoculation spatula (Laboquimia). This material was poured into 15 ml Falcon tubes (Life Sciences Products) and kept at 4 °C. From these samples, the DNA extraction was carried out with an Exgene Plant SV mini kit (Gene All Products) according to manufacturer’s protocols. Two elutions were performed with nuclease-free water, each with a volume of 50 μL to obtain a higher DNA yield. Elutions were concentrated to approximately 25 μL using a SpeedVac concentrator (DNA120 Savant kit, Thermo Scientific, USA). DNA was quantified on a Qubit fluorometer (Thermo Fisher Scientific, USA) and stored at 4 °C until sequencing.

#### 2.4.2. CµS-sampler samples.

The PBS was filtered using a Swinnex Filter Holder (25 mm) equipped with 0.22 µm sterile Millipore filters (Merk). The filters were stored at 4 °C until they were processed for DNA extraction, purification, and subsequent massive sequencing (Fig 1B).

DNA extraction from fungi collected in PBS with CµS-Sampler. Six samples of PBS from the air of each evaluated area were processed to perform the metagenomic analysis. The DNA extraction was performed using the Gene All, Exgene Plant SV mini kit according to manufacturer’s protocols (Gene All, Biotechnology Co., Ltd). Two nuclease-free water elutions were performed, each with a volume of 50 μL, to obtain a higher yield of DNA. The elutions were concentrated at a volume of approximately 25 μL with a SpeedVac concentrator (DNA120 Savant, Thermo Scientific, USA) and stored at 4 °C until sequencing.

### 2.5. Illumina sequencing

Illumina DNA sequencing of fungi samples collected from the air with the AVPS and CµS-Sampler was carried out as follows: The DNA was quantified using a Qubit fluorometer (Thermo Fisher Scientific, USA), and its purity was verified by spectrophotometry with the nanodrop MODELO and sent to the Instituto Nacional de Medicina Genómica (INMEGEN) to construct ITS libraries. For the fungal ITS2 region, the protocol described by [32] was followed (2011), but using primers ITS3_KY02 [33]/ ITS4 [34] and an alignment temperature of 52° C. The prepared libraries were sequencing on the Illumina NextSeq 1000/2000 using a 600-cycle kit in a P1 flow cell. The bioinformatics analysis was performed at the Unidad Universitaria de Secuenciación Masiva y Bioinformática, Instituto de Biotecnología (UUSMB, IBT, UNAM). The sequencing raw data and sample information are publicly available in the NCBI BioProject database under the PRJNA1165756 ID, Submission ID: SUB14752941

### 2.6. Bioinformatic analysis

#### 2.6.1. Sanger sequencing.

ITS and TEF sequences were edited with the Geneious Prime software. A taxonomic approximation was conducted using the BLAST algorithm with default parameters to compare the query sequence against sequences deposited in the nucleotide NCBI database. The highest-scoring alignments were considered to represent the most likely taxonomic assignations S1 Table.

#### 2.6.2. NGS analysis.

The reconstruction of the original ITS amplicon region was done by overlapping the pair-end reads using the software Flash version 1.2.11 [35]. The resulting merged paired sequences were used for the taxonomic annotation process, performed with the Parallel-META program version 2.4.1, [36] against the Metaxa2 database version 2.1.1 [37] as previously described by [38]. Statistical analyses and plotting were performed using R Statistical Software [39]. Manipulation of data, barplots and diversity indexes were done with the phyloseq package [40], rarefaction curves were built using the ranacapa package [41], PCA analysis was performed and plotted using the ade4 package [42], heatmaps were plotted with ggplot2 package [43] and Venn diagrams with given package [44] by ggplot2.

## 3. Results

### 3.1. Sequencing data

The DNA concentration and purity obtained from the samples were suitable for sequencing. All the samples presented a base quality equal to or greater than Q20 (99% accuracy) and no adapters were detected.

The characteristics of the metagenomic analysis are shown in Table 1. Samples obtained with the AVPS had a total maximum sequenced reads of 5161093 (raw sequences) obtained from F2. On the other hand, with the CųS-Sampler, the maximum sequenced reads were 7974508 obtained in F2, these were validated after quality filtering and obtaining post-fusion reads for subsequent analysis. Outside the hospital (OH), the maximum readings were 4809321 obtained with the AVPS and 4124927 with the CųS-Sampler (Table 1). Regarding sequence identity, the total taxonomic annotations at the genus level were obtained with the CųS-Sampler, mainly in F2 (632) and F1 (626). The present study obtained more sequenced readings with the CμS-Sampler device (Table 1).

**Table 1:** Descriptive characteristics of the metagenomic analysis (ITS).

Characteristic	ITS							
	AVPS				CųS			
	OH	F1	F2	EU	OH	F 1	F 2	EU
Raw reads	4809321	4947664	5161093	3451865	4124927	4975702	7974508	3975092
Post-merging reads	4614796	4731753	4963119	3347901	3941225	4829228	7690388	3864529
Total taxonomy annotations at genus level	213	260	209	195	617	626	632	485
Unclassified taxa at genus level	0	0	0	1	2	1	3	2
Classified taxa at genus level	213	260	209	194	615	625	629	483
Unique Known Genera	24	47	27	25	56	46	65	20

**Fig 2:**
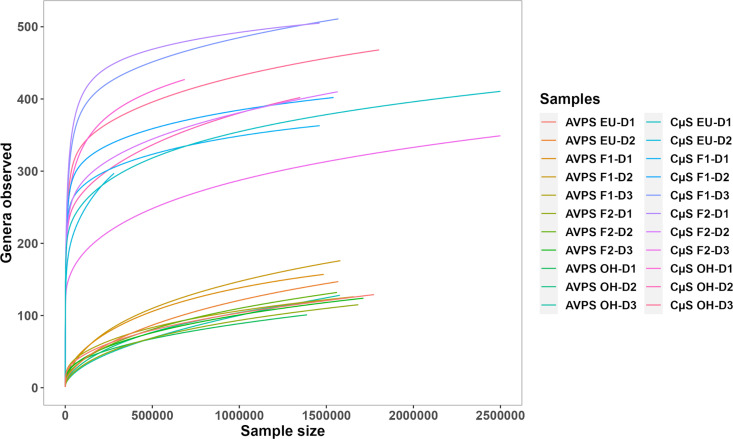
Rarefaction curves of the fungal genera were observed for each sampling area. The legends correspond to the samples obtained with the AVPS (lower richness of genera), and those on the right side indicate the bacteria samples collected with the CųS-Sampler (higher richness and density of genera) in each monitored hospital area.

The bioinformatics analysis of the ITS (fungal) indicated through the rarefaction curves that the sampling of the bioaerosols and the detection of DNA allowed reaching the correct sampling force to carry out the molecular analysis (Fig 2). Rarefaction curves estimate the expected number of species for a given sample size based on a hypergeometric distribution. In this figure, most samples are close to reaching a plateau, indicating that the sequencing depth obtained is adequate for our analysis.

### 3.2. Fungal community composition

**Fig 3:**
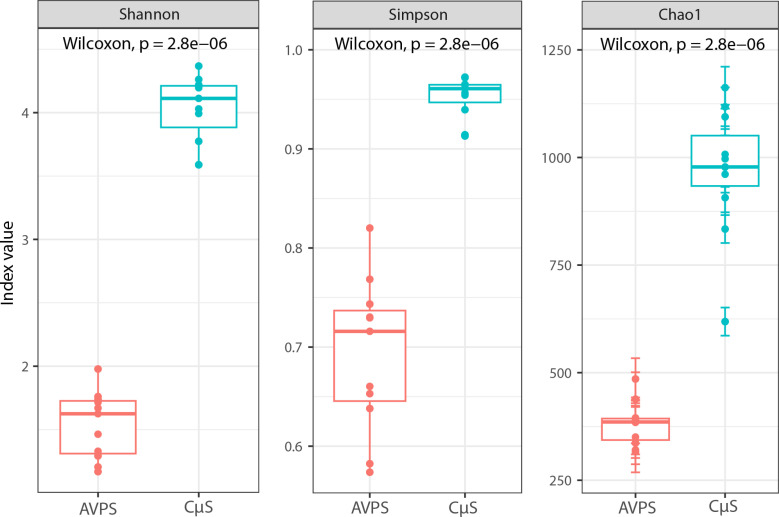
Alpha indices (Shannon and Simpson and Chao) from the total samples collected with the AVPS and the CųS-Sampler samplers, and the statistical values obtained with the Wilcoxon test to determine differences between both air samplers.

To better describe and compare communities, we used metrics adapted to metagenomics. The Shannon and Simpson indices (Gini-Simpson index) and Chao are alpha (α) diversity metrics that assess the diversity of local communities. These indices indicated that greater abundance, dominance, and species richness were obtained with the CųS sampler than with the AVPS sampler (Fig 3).

**Fig 4:**
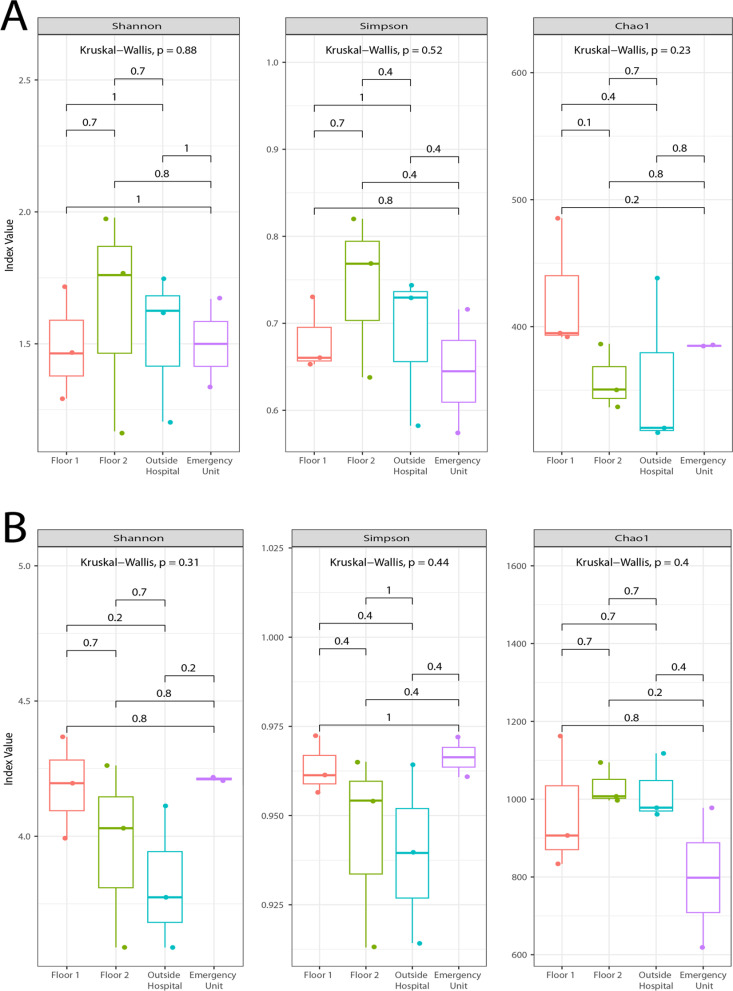
Alpha indices (Shannon and Simpson) and Chao index from the samples collected with the (A) AVPS and the (B) CųS-Sampler samplers in the different sampled areas of the hospital and outside; and the statistical values obtained with the Kruskal Wallis test to determine differences between the sampled areas.

Samples collected with the AVPS (Fig 4A) and CųS-Sampler (Fig 4B) samplers were compared for each index. The nonparametric Kruskal-Wallis test was used to determine possible significant differences between the sampled areas of the hospital (OH, F1, F2, and EU) for each diversity.

According to the Shannon and Simpson diversity indices and the Chao index, the fungal diversity data collected with both the AVPS (Fig 4A) and the CųS-Sampler (Fig 4B) did not show significant differences (Kruskal-Wallis test) among the sampled areas. Values less than 2 obtained with the Shannon index for AVPS indicate low diversity, in contrast to those observed for CųS-Sampler, which reached values greater than 4, indicating high diversity of recorded species.

The KRONA graph shows all phyla and genera recorded with both samplers in each sampled area, see Supplementary Materials (S1 Fig)

#### 3.2.1. Phyla present in the air sampled from the different areas of the hospital and outside.

**Fig 5:**
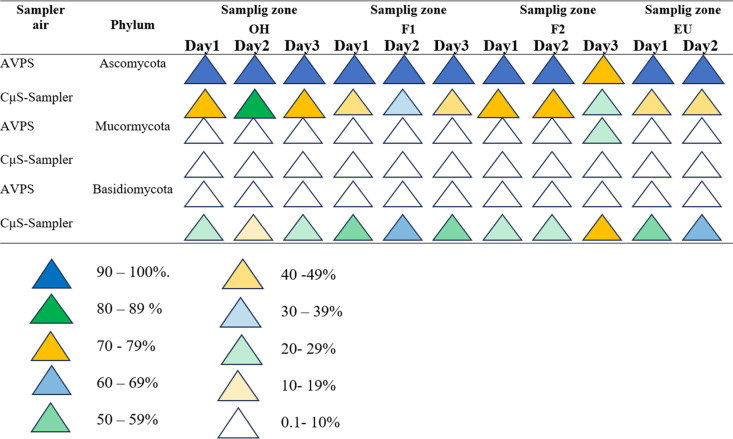
Percentages per day of the main fungal phyla registered with the AVPS and CųS-Sampler in each hospital area.

The fungi collected using the AVPS predominantly belonged to the phylum Ascomycota, with relative abundance ranging from 90% to 100% both inside and outside the hospital. Basidiomycota followed with a relative abundance of 1% to 10%, and Mucormycota showed a relative abundance between 4% and 26% within the hospital environment (Fig 5).

In contrast, samples obtained using the CμS-Sampler revealed a relative abundance of Ascomycota ranging from 39% to 72% in F1 and F2, respectively, and 73% to 82% outside the hospital. Followed by, Basidiomycota exhibited a higher relative abundance when using the CμS-Sampler compared to the AVPS, with values between 54% and 61% in F1, while outdoor values samples fluctuated between 18% and 27% (Fig 5).

#### 3.2.2. Main families of fungi identified in the air in the different areas of the hospital and outside.

Inside the hospital, the highest relative abundances obtained with both samplers corresponded to Dotiodeomycetes (F2 and F1), Eurotiomycetes (Eu, F1, F2), Sordariomycetes (Eu, F2, F1) and Leotiomycetes (F2 and F1), as well as Agaricomycetes, which were isolated mainly with CųS-Sampler. Mucormycetes were only isolated with AVPS in F2 and EU. Some Dotiodeomycetes, Eurotiomycetes and Agaricomycetes were isolated outside the hospital (S1 Fig).

#### 3.2.3. Main genera of fungal identified in the air in the different areas of the hospital and outside.

**Fig 6:**
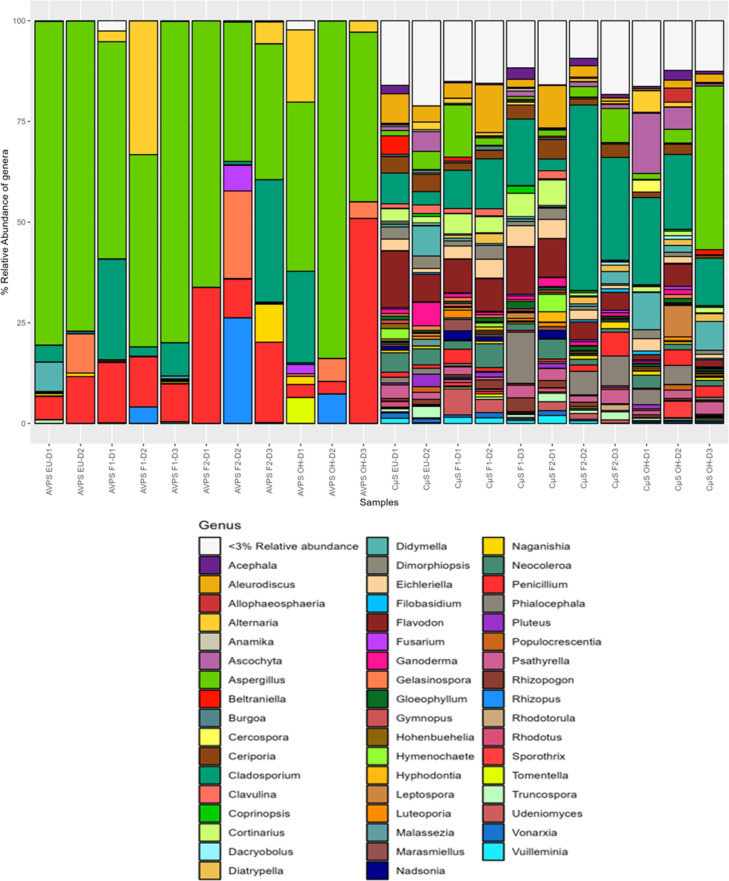
Relative abundance of fungi genera collected with AVPS and CųS-Sampler.

(Fig 6) highlights significant differences in the diversity and distribution of fungal genera between the two collection methods, AVPS and CμS-Sampler. The samples collected using the AVPS method tend to be dominated by a few genera, such as *Aspergillus*, *Alternaria,* and *Penicillium,* indicating lower fungal diversity. In contrast, the samples collected using the CμS-Sampler show a greater diversity, with a wider range of genera present in more balanced proportions. This suggests that the CμS-sampler may capture a broader variety of fungal genera (*Aspergillus, Cladosporium, Rhizopogon* and *Aleurodiscus*, among others) compared to the AVPS, which appears to be more selective for certain dominant genera (EU, F1, F2 and OH) (S1 Fig).

**Fig 7:**
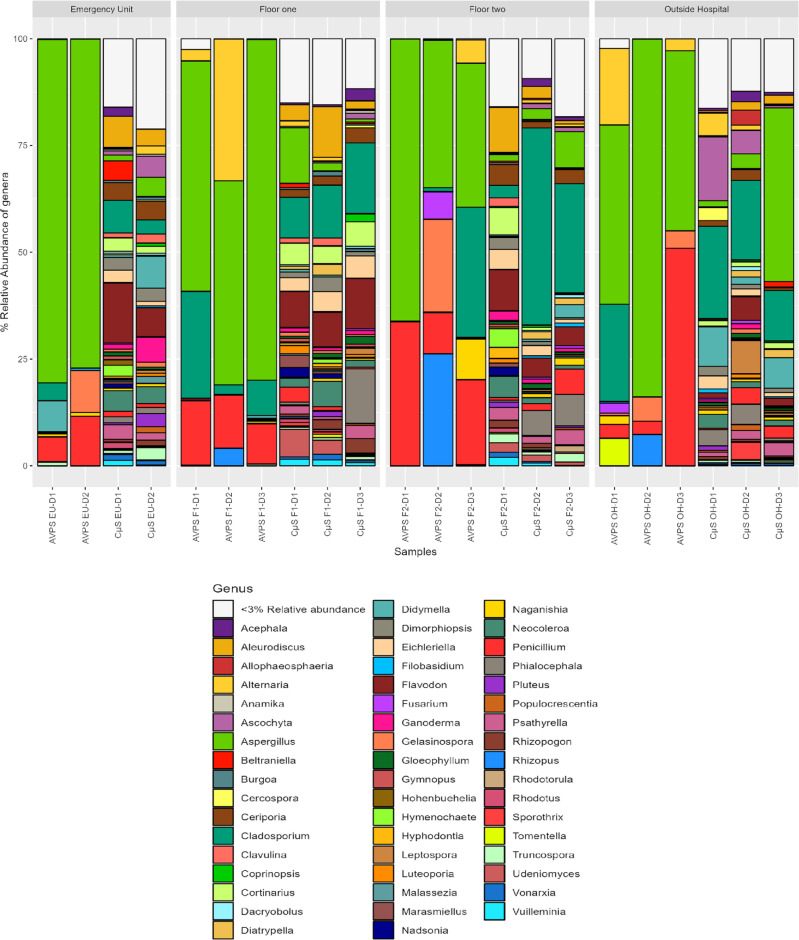
Relative abundance of the main fungal genera collected with AVPS and C μS-Sampler air samplers in the different sampling areas inside and outside the hospital.

**Fig 8:**
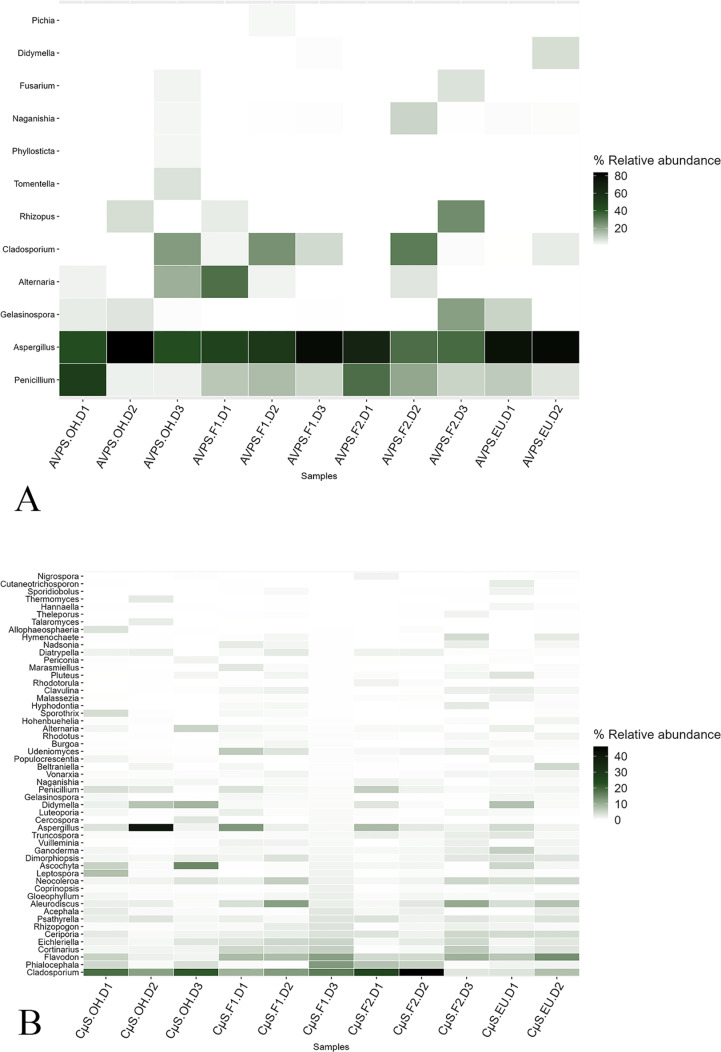
Relative abundance of the main fungal genera collected with air samplers in the different sampling areas inside and outside the hospital. A) AVPS, B) CμS-Sampler. AVPS sample suffix = Samples obtained with the AVPS collector. CųS-Sampler sample suffix = Samples obtained with the CuS-Sampler collector.

Greater diversity is observed in the samples collected with CμS-Sampler, in which fungi such as *Aspergillus, Cladosporium, Rhizopogon* and *Aleurodiscus*, among others, were isolated, while with AVPS the predominant genera were *Aspergillus, Penicillium, Alternaria,* and *Cladosporium*.

(Fig 7) shows the relative abundance of the main fungal genera collected with AVPS and CųS air samplers in the different sampling areas inside and outside the hospital and (Fig 8) shows a heat map of the relative abundance of the main genera of fungi collected with A) the AVPS and B) the CμS-Sampler in the different sampling areas inside and outside the hospital.

The highest percentages of fungi collected with the AVPS in the EU, on Floors 1 and 2 and even outside the hospital, corresponded to fungi of the genus *Aspergillus*, followed mainly by *Penicillium, Cladosporium,* and *Alternaria*. It is observed that the relative abundance of fungal genera collected varied by area and day within the hospital. For instance, *Aspergillus* was detected in all hospital areas, with notably higher relative abundances (approximately 80%) observed on day 1 in both F2 and the EU; on day 2 in the EU and OH; and day 3 on F1. Meanwhile, *Cladosporium* exhibited a higher relative abundance on day 2 on F2, and *Alternaria* predominated on day 1 on F1.

In contrast, sampling with the CμS-Sampler revealed a greater diversity of fungal genera predominating *Aspergillus, Cladosporium,* among many others. *Aspergillus* was predominant on day 2 in the OH area. Also, *Aspergillus* was collected in all the areas evaluated, varying wildly in abundance (Fig 7, Fig 8, S1 Fig).

Table 2 shows the main genera of airborne fungi collected with the AVPS and CųS-Sample during the sampling days.

**Table 2:** Relative abundances of the most abundant fungal genera collected with the AVPS and with the CųS-Sampler (S1 Fig).

Sampler AVPS	% Out of Hospital			% Floor 1			% Floor 2			% Emergency Unit	
Fungi	Day 1	Day 2	Day 3	Day 1	Day 2	Day 3	Day 1	Day 2	Day 3	Day 1	Day 2
Aspergillus	42	84	42	48	54	80	66	34	35	77	80
Penicillium	51	3	3	12	15	9	34	20	10	12	6
Cladosporium	--	--	23	2	22	8	--	30	--	---	4
Alternaria	3	--	18	33	3	--	--	6	--	--	--
CųS-Sampler	Day 1	Day 2	Day 3	Day 1	Day 2	Day 3	Day 1	Day 2	Day 3	Day 1	Day 2
Aspergillus	3	41	1	13	2	0.7	8	3	2	4	1
Penicillium	4	3	--	4	0.9	---	6	2	0.6	0.9	1
Cladosporium	14	12	22	10	12	17	26	46	3	3	8
Alternaria	2	5	--	1	0.8	--	0.6	0.8	--	2	--

Table 2 clearly shows that *Aspergillus and Penicillium* were abundant inside and outside the hospital. In addition, *Cladosporium* reached its maximum levels in F1 and F2 and in OH.

**Fig 9:**
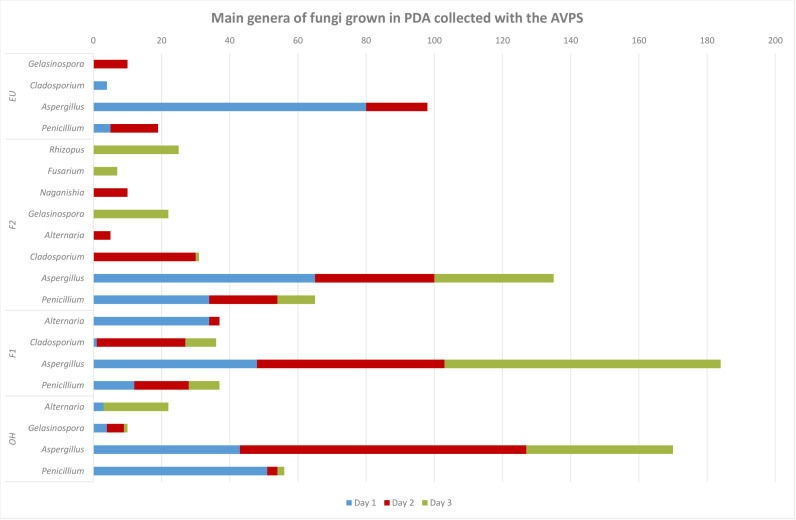
Principal Component Analysis (PCA) showing the clustering of the main genera of airborne fungi, collected with the AVPS in various intra- and extra-hospital areas, and grown on potato dextrose agar (PDA). AVPS sample suffix = Samples obtained with the AVPS collector. CųS-Sampler sample suffix = Samples obtained with the CuS-Sampler collector.

The Principal Component graph (PCG) shows the grouping of the main genera of fungi collected with the AVPS and the CμS-Sampler in the different sampled areas. It can be observed that the fungi collected with the CμS-Sampler present a greater variability or diversity of genera compared to those collected with the AVPS, whose genera are concentrated in only two quadrants. In comparison, those collected with the CμS-Sampler cover the four quadrants of PCG, observing greater precision and capacity for detecting fungi present in the samples even with low abundance (Fig 9).

It is observed that the fungal genera most frequently collected with both sampling equipment corresponded to species of *Aspergillus* and *Penicillium*.

**Fig 10:**
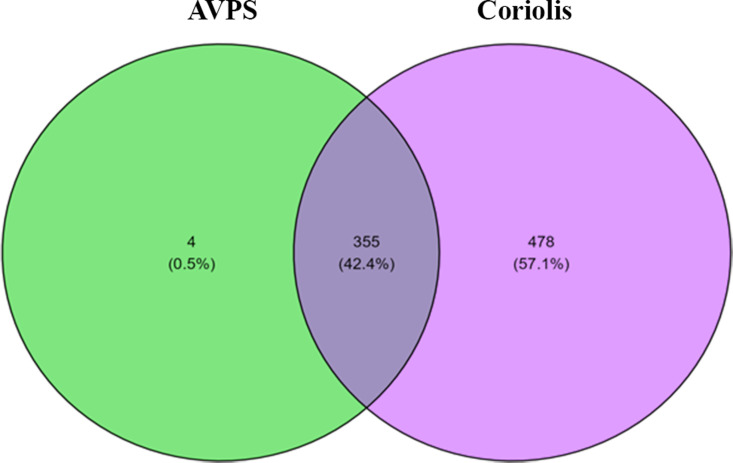
Venn diagram showing the percentage of fungi genera registered with AVPS and C μS-Sampler.

Venn diagram showing the percentage of fungi genera registered with AVPS and CμS-Sampler is shown in (Fig 10).

**Fig 11:**
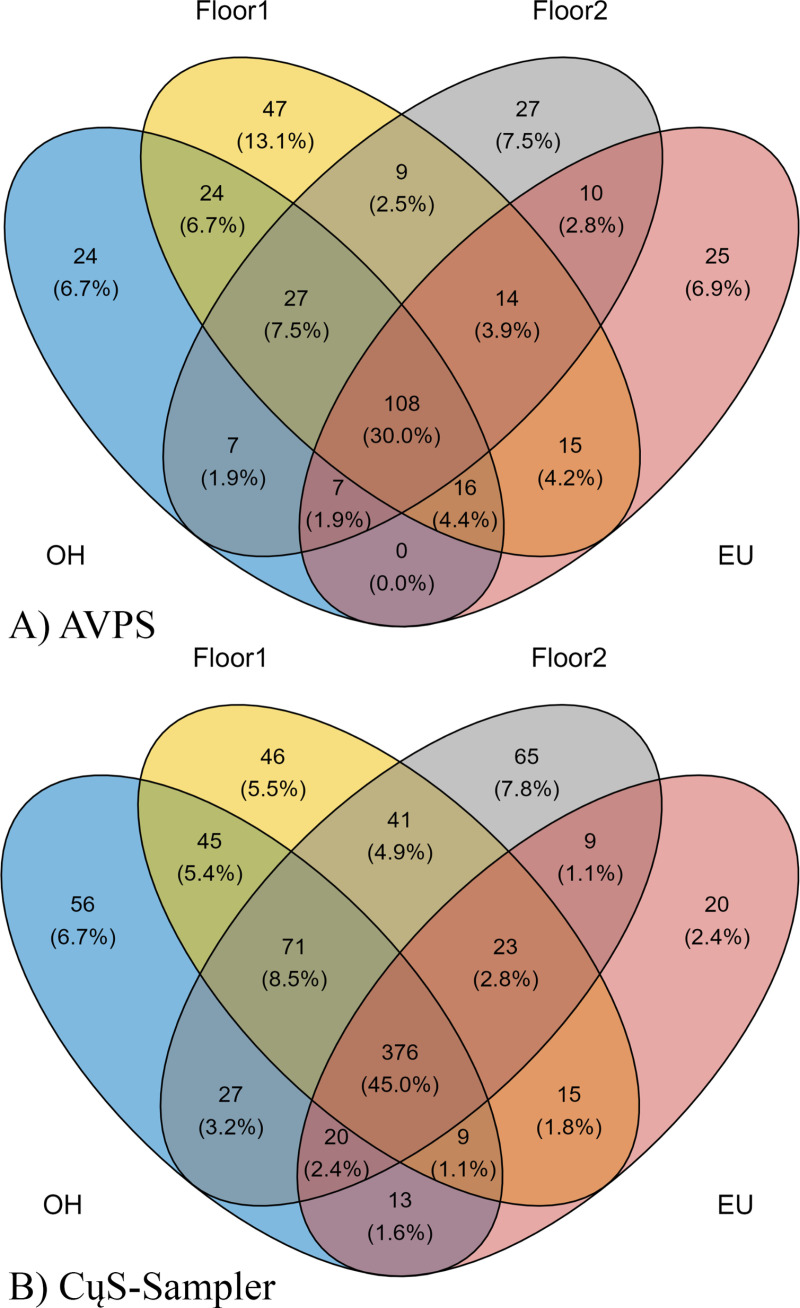
Venn diagram showing the relative abundance and number of taxa at genus level isolated with A) with AVPS and B) with the CμS-Sampler, for each of the sampled areas inside and outside the hospital. AVPS sample suffix = Samples obtained with the AVPS collector. CųS-Sampler sample suffix = Samples obtained with the CuS-Sampler collector.

The CμS-Sampler collected 57.1% of the fungal taxa not captured by the AVPS. The AVPS, on the other hand, managed to collect only 0.5% of fungi other than those obtained with the CμS-Sampler, representing four different taxa. The fungal genera collected by both devices accounted for 42.4% of the airborne fungi.

(Fig 11) shows the relative abundance (%) and number of taxa at genus level (G) isolated with A) AVPS and B) CμS-Sampler in each of the sampled areas inside and outside the hospital.

**Fig 12:**
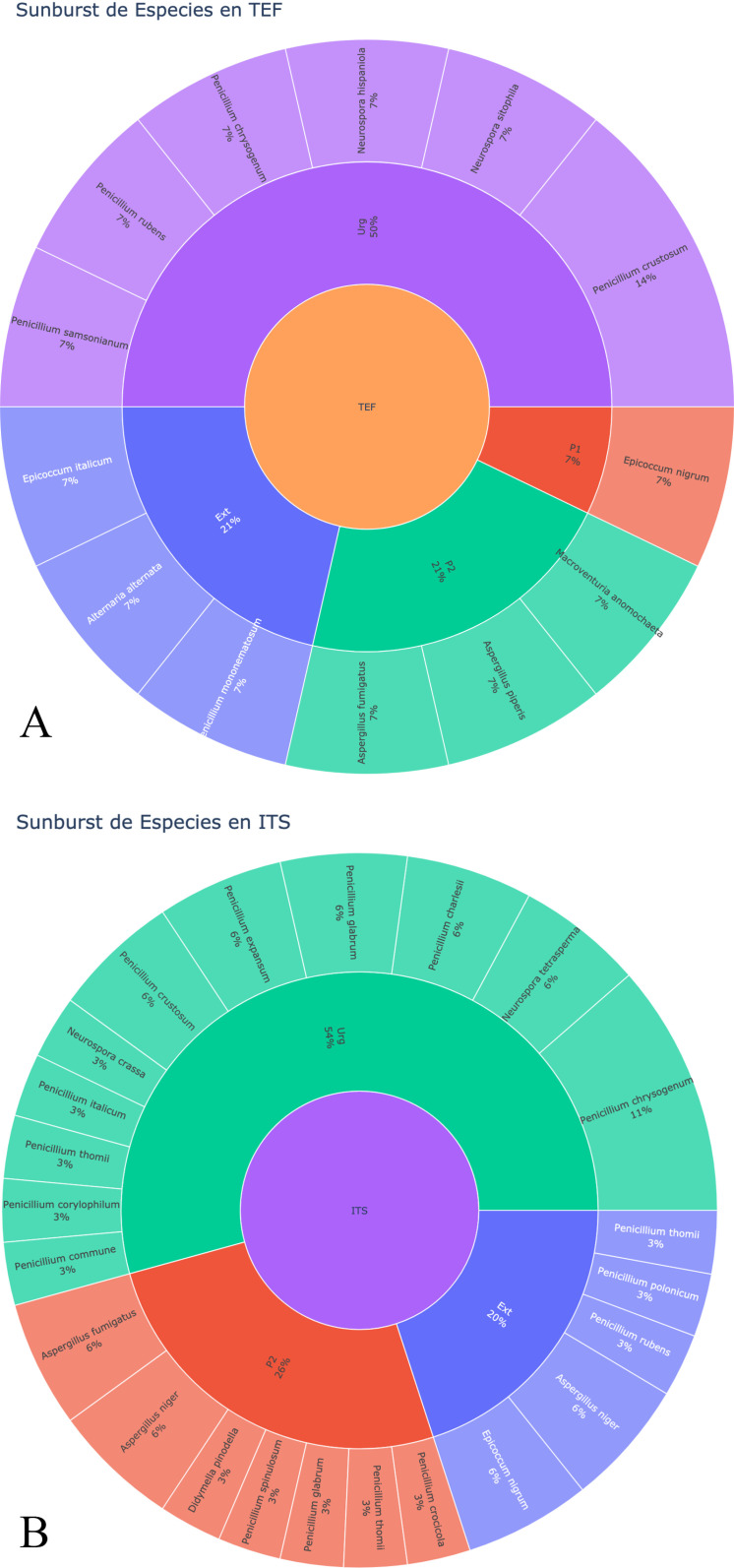
Main airborne fungal species detected in the study inside and outside the hospital. The graph represents the predominant airborne fungal species isolated and identified, A) using the sequenced TEF regions; B) using the sequenced ITS regions.

Our data show that the AVPS collected the most taxa recorded in F1 (13.1% = 47 G), followed by F2 (7.2% = 27 G), EU (6.9% = 25 G), and OH (6.7% = 24G). Notably, with the CμS-Sampler, 57.8% was obtained (equivalent to 478 G). Of them, the highest percentage was recorded in F2 with 7.8% (65 G), followed by those recorded in OH with 6.7% (56 G), F1 with 5.5% (46 G), and the EU with 2.4% (20 G). When relating the genera obtained between the sampled sites, matches were found between OH-F1 at 5.4% (45 G), F1-F2 at 4.9% (41 G), OH-F1-F2 at 8.5% (71 G), and the highest percentage of matching fungal genera (45%) between F1-F2-OH-EU corresponded to 376 G (Fig 12).

In summary, the CμS-Sampler sampler demonstrated its efficacy by isolating the largest number of fungal genera, a significant finding that underscores its potential in our research. With the AVPS, most of the genera obtained were recorded on Floors 1 and 2, followed by the Emergency Unit and Outside the Hospital. With the CųS, the largest number of genres were isolated on Floors F2, F1, and Outside the Hospital.

#### 3.2.4. Main species of fungi identified in the air in the different areas of the hospital and outside.

A total of 27 fungal species were identified using the markers TEF (Translation Elongation Factor) and ITS (Internal Transcribed Spacer). In the EU, 50% of the species were identified with TEF and 54% with ITS. In F2, 21% were identified with TEF and 26% with ITS. Outside (outside the hospital entrance, OH), 21% were identified with TEF and 20% with ITS (S1 Fig), (Table 3).

**Table 3:** Airborne fungal species isolated inside and outside the monitored hospital, identified using sequences from the TEF and ITS regions, along with their potential effects on human health.

Specie Name 27 species of fungi	TEF %	ITS %	Health Effects	Reference
Total percentages by area	UE 50 F1 7 F2 21 OH 21	UE 54 F1 — F2 26 OH 20%		
*Alternaria alternata*	7 (OH)	—	It can trigger allergic reactions in some sensitive individuals, such as allergic rhinitis, allergic conjunctivitis, and asthma. It has also been associated with infections in immunocompromised individuals, including skin, sinus, and bronchitis. As well as known as trigger allergic respiratory diseases	[45–48]
*Aspergillus fumigatus*	7 (F2)	6 (F2)	It is a filamentous fungus and a crucial opportunistic pathogen in humans and people with weakened immune systems. The infections it causes are grouped into different forms of aspergillosis, depending on the site of infection and the status of the host’s immune system. Exposition to *Asapergilus* fungi causes Invasive pulmonary aspergillosis, Allergic bronchopulmonary, Aspergilloma (fungal ball), A. Cutaneous, and sinusitis by Aspergillus. Main agent of aspergillosis in immunocompromised patients. Colonization and invasion are accompanied by allergic reactions. Typically causing inhalation mycosis	[49,50]
*Aspergillus niger*	—	6 (F2, OH)	*Aspergillus* is a fungus present in various environments, such as air and soil, and it usually doesn’t affect healthy individuals. However, in hospitals, where many patients have weakened immune systems, it can become a serious health risk. It can cause pulmonary aspergillosis, sinusitis, otitis, hay fever, systemic mycosis.	[51–53]
*Aspergillus piperis CBS 112811*	7 (F2)	—	*A. pipers* is a species known for its ability to cause infections in humans, especially those with weakened immune systems. The risk posed by this species of this genus should not be ignored. It is widespread in construction or renovation works and ventilation systems.	[53,54]
*Didymella pinodella*	—	3 (F2)	It is a phytopathogenic fungus of legumes. As for its allergenicity, there are no studies that directly link it to allergic reactions in humans. However, as with other phytopathogenic fungi, handling and prolonged exposure to spores can potentially cause respiratory symptoms in sensitive people or those predisposed to allergies.	[55]
*Epicoccum nigrum* *Epicoccum italicum*	7 (F1)	6 (OH)	They are a health risk in hospital environments, mainly through allergic reactions and, to a lesser extent, as opportunistic pathogens in immunocompromised individuals. The main risk factor is exposure to airborne spores, especially in poorly ventilated or humid environments. Its effects are known as triggering allergic respiratory diseases.	[47,48,56]
	7 (OH)	—		
*Macroventuria anomochaeta*	7 (F2)	—	It is a phytopathogenic fungus that affects certain plants, such as species of the Rosaceae family. The spores can trigger respiratory symptoms in sensitive individuals, contributing to respiratory allergies in certain circumstances.	[57,58]
*Neurospora crassa*	—	3 (UE)	*Neurospora sitophila* has been linked to cases of occupational asthma in workers exposed to cork dust and coffee grounds in food and wood processing industries. The spores of this fungus are easily dispersed and can induce respiratory symptoms in sensitive individuals.	[59]
*Neurospora hispaniola*	7 (EU)	—		
*Neurospora sitophila*	7 EU)	—		
*Neurospora tetrasperma*	—-	6 (EU)		
*P. charlesii* *P. commune* *P. crocicola* *P. crustosum* *P. corylophilum*	—	6 (EU)	In general, *Penicillium* spores are common indoors and outdoors, and are potential allergens and responsible for respiratory allergies in sensitive individuals.	[60,61]
	—-	3 (EU)		
	—	3 (EU)		
	14 (UE)	6 (EU)		
	—-	3 (EU)		
*Penicillium chrysogenum*	7 (UE)	11 (EU)	*P. chrysogenum*, best known for its use in penicillin production, also has implications for human health. Some strains can be opportunistic pathogens and cause infections, especially in people with weakened immune systems. They also cause Allergic reactions and mycotoxicosis.	[60,62,63]
*Penicillium expansum*	—	6 (EU)	*P. expansum* can cause allergic reactions and induce opportunistic infections in immunocompromised individuals. Exposure to its mycotoxins, such as patulin, can also be a health concern if ingested in contaminated food.	[64]
*Penicillium glabrum*	—	6 (EU); 3 (F2)	*P. purpurogenum* is an opportunistic fungus that can affect human health, especially through allergic reactions or infections in immunocompromised individuals. Exposure to its spores or mycotoxins in poorly ventilated or high-humidity environments is the main risk for healthy individuals.	[65,66]
*P.italicum*	—	3 (EU)	In general, spores of fungi of the *Penicillium* genus and its various species are common indoors and outdoors and can cause respiratory allergies in sensitive people.	[60]
*P. mononematosum*	7 (OH)	—		
*P. polonicum*	—	3 (OH)		
*P. rubens*	7 (EU)	3 (OH)		
*P.samsonianum*	7(EU)	—		
*P.spinulosum*	—	3 (F2)		
*P. thomii*	—	3 (EU, F2, OH)		

Emergency Unit (EU); Floor one (F1); Floor two (F2); Out of the Hospital (OH)

(Fig 12) shows the percentages of fungal species isolated from the air, grown in the culture medium used with the AVPS sampler, and identified using the BLAST (Basic Local Alignment Search Tool) system from sequences obtained from specific genes, such as the TEF (Translation Elongation Factor) and the ITS (Internal Transcribed Spacer) region. Most of these species are not only an indicator of air quality, but also a warning sign that can have a direct impact on the health and safety of the most vulnerable patients (Table 3).

### 3.3. Microclimatic conditions and suspended particles in airborne indoor and outdoor hospital

Table 4 shows the microclimatic conditions and air quality inside and outside the hospital during bioaerosols sampling. The average temperature in F1 and F2 fluctuated between 26 and 27 °C, while in the EU and OH, an average of 24 °C was recorded. The highest relative humidity values were found in these last two areas (41.9 and 47.3%, respectively) (Table 4).

**Table 4:** Air quality conditions (airborne particles), temperature, and relative humidity recorded with the airborne particle counter (model 8306, Particles Plus brand) in the outside and inside air of the hospital.

Zona		Temperature ºC	Relative Humidity %	PM^*^ 0.5µm µg/m^3^	PM^*^ 1.0µm µg/m^3^	PM^*^ 2.5µm µg/m^3^	PM^*^ 3.0µm µg/m^3^	PM^*^ 10.0µm µg/m^3^	TPM^**^ µg/m^3^
Outside Hospital	Mean	24.8	41.9	8.7	11.4	17.2	35.0	66.8	188.4
	Interval	22-29.4	34-49	0.76-13.6	1.4-18.7	4.9-28.1	13.5-72.8	24.8-208	47.1-753.0˚
	Standard deviation	1.66	5.16	3.00	3.90	5.37	13.08	33.22	118.70
Floor One	Mean	26.0	43.5	5.9	9.4	24.1	58.6	101.6	303.3
	Interval	20.4-27.0	37-58	1.55-18.3	4.42-23.9	9.2-73.1	24.1-244	43.8-553	129.6-1642
	Standard deviation	1.35	4.20	4.93	5.56	10.16	28.52	53.11	174.98
Floor Two	Mean	27.00	39.8	8.1	12.1	22.0	44.1	73.3	195.0
	Interval	24.4-27.9	37-43	2.2-22.3	3.8-31.6	7.2-44.9	14.02-73	25.7-130	79.6-653.0
	Standard deviation	.91	1.25	7.20	9.67	11.31	16.58	22.02	75.31
Emergency Unit	Mean	24.00	47.3	1.8	3.3	9.9	31.4	6.8	235.80
	Interval	20.1-24.9	44-56	0.79-4.1	2.08-6.35	6.32-19.2	15.3-64.4	30.16-135	80.02-591.1
	Standard deviation	1.14	2.7	.78	1.12	3.00	11.29	25.80	105.75

* PM = Particulate Matter

** TPM= Total Particulate Matter

Regarding the concentration of particles quantified in the air, from 0.5 to < 2.5 µg/m^3^ were obtained in the EU, and the highest values were in F1 and F2. Regarding particles of 5 and 10 µm, these were recorded on both monitored floors, presenting the highest values in F1. Likewise, the highest value of TPM was recorded in F1 (303 µg/m^3^ followed by EU (235.8 µg/m^3^), (Table 4).

## 4. Discussion

As a result of the assessment of the indoor and outdoor air quality of the hospital studied, we found the presence of a high diversity of fungi. The alpha and Chao indices showed that there was a higher abundance, dominance, and richness of fungal genera obtained with the CųS-Sampler compared to the AVPS. It is essential to highlight that the use of both samplers is complementary, allowing for the identification of a greater abundance and diversity of fungi in the air of the hospital environment and surrounding areas.

In this study, samples collected with the AVPS did not show significant differences in the abundance and richness of genera between the sampled areas, with low diversity being observed. In contrast, fungal samples obtained with the CųS-Sampler indicated that, although no significant differences were found in the Shannon and Simpson indices between the areas, values greater than 2 suggested that the highest diversity and richness of fungi was found inside the hospital compared to outside. Furthermore, using the CųS-Sampler, high diversity was observed in the Emergency Unit (EU), in addition to that recorded on floors one and two.

Notably, the areas with the highest activity were located on floors F1 and F2 due to the presence of patients, health personnel, cleaning staff and relatives. Likewise, although access to the UE is restricted to patients and a minimum of health personnel, such as doctors and nurses, there is a constant flow of activity, with patients entering and leaving other areas of the hospital, which promotes the dispersion of spores in the environment. Likewise, outside the hospital, there are many patients, relatives, health personnel, street vendors and vehicle traffic, which allows the dispersion of particles in the air, such as individual fungal propagules, in aggregates or attached to aeroparticles.

Our rigorous study found that the main fungal groups belonged to the species Ascomycetes, followed by Basidiomycetes and, in lesser abundance, Mucormycetes. With the two samplers used, the AVPS and the CųS-Sampler, Ascomycetes dominated inside and outside the hospital, while Basidiomycetes fungi were isolated mainly with the CųS-Sampler since the samples were collected in liquid and processed by molecular techniques, sequencing the DNA and determining the main fungi present in the air.

Families, genera and species reported as potentially pathogenic or allergenic were identified, a finding that coincides with several studies that have also highlighted the presence of these phyla in the air. For example, [67] in a comprehensive study of bacteria and fungi in bioaerosols from specialized hospitals in eastern China, found that the two most abundant fungal phyla were Ascomycota (51.2%) and Basidiomycota (26.2%), with 77.4% of the total readings. Mycophaerellaceae, Aspergillaceae and Physalacriaceae were the most abundant of the ten families recorded. These findings are consistent with those recorded in this study in which fungi of the Aspergillaceae class of the Eurotiomycetes family were found, as well as Agaromyctes of the Physalacriaceae family and some genera of the Dothideomycetes class. Furthermore, [46] reported that in a study on allergic reactions of people exposed to fungi, it was found that type 1 hypersensitivity reactions occurred upon exposure to approximately 80 types of fungi, predominantly Ascomycota species.

The principal genera collected inside and outside the hospital using the AVPS system were *Aspergillus, Penicillium, Cladosporium,* and *Alternaria.* In contrast, the CųS system predominantly isolated *Aspergillus* and *Cladosporium.* The most abundant genera, according to both sampling methods, were *Aspergillus*, which showed the highest percentages in areas F1, F2, and in the Emergency Unit, as well as outside the hospital. This was followed by *Penicillium* species, which were isolated mainly inside the hospital, with the presence of both genera in the Emergency Unit being notable. Likewise, *Cladosporium* was present both inside and outside the hospital.

It is crucial to consider the health risk posed to patients by exposure to fungal spores in hospital air. Small conidia, such as *Aspergillus* and *Penicillium*, are usually deposited in the upper respiratory tract, while smaller spores and fungal fragments (<1 µm) can reach the lower respiratory tract. Both spore and fungal extracts can cause asthmatic allergic reactions in the early and late stages in allergic individuals [68,69].

More than 80 fungal genera have been associated with allergic respiratory symptoms; However, only a few, such as *Alternaria, Cladosporium, Aspergillus* and *Penicillium*, have been studied as causes of allergic diseases [46,48,70,71].

The types of fungi identified in the air of the hospital evaluated are consistent with previous research on airborne contamination in hospitals, where several species of filamentous fungi have been identified. For example, [72] analyzed air samples from 83 sites and found that *Cladosporium, Penicillium, Aspergillus,* and *Alternaria* were the most common genera. Similarly, a study by [73] in a pediatric hospital unit reported that *Cladosporium, Alternaria, Penicillium, Aspergillus,* and *Acremonium* were the most prevalent genera.

Of the 27 fungal species identified inside and outside the hospital, 15 were isolated in the Emergency Unit and nine on the second floor, while eight were found outside. *Aspergillus fumigatus, A. piperis* and *A. niger* were detected on the second floor, the latter also being isolated outside the hospital. These species are common in high-activity environments, as observed during the morning hours in the hospital assessed. In addition, the remodeling of the hospital and the construction of a new adjacent building caused high dust concentrations, as also indicated by Total Particulate Matter (TPM) measurements, which ranged from 174.9 to 303.3 µg/m³ inside the hospital and 188.4 µg/m³ outside. The particle size range evaluated, from 0.5 µm to 10.0 µm, is directly relevant to the presence of fungi, as many fungal spores are found within this range. Although spore size varies by species, a significant proportion is between 1 µm and 10 µm. This means that the particle measurements performed can be associated with the presence of airborne fungal spores. Several studies have reported airborne microparticles and nanoparticles in urban areas associated to fungal spores such as *Aspergillus* and to pollen grains, and delving into the health impacts by exposure to these particles and bioaerosols [6,26,74,75]. The increase in aspergillosis cases has been associated with hospital construction, as they increase the concentration of spores in the air, including *Aspergillus piperis,* a species that we also isolated in our study and that has been reported as common in construction environments [76]. Likewise, the presence of *A. fumigatus* in the hospital coincides with a study in which the demolition of a building adjacent to a hospital was carried out, where the concentrations of thermotolerant fungi such as *A. fumigatus* were measured before and after the demolition, outside and inside of the hospital, finding that after the demolition, there was a significant increase in this fungus, mainly in the outside air of the hospital [77]. It must also be considered that the temperature conditions (between 24 ºC and 27 ºC) and relative humidity (39% and 47%) recorded inside our hospital were optimal for the development of these fungi in various substrates, such as organic matter, walls, plastics, ventilation pipes, air conditioners and humidifiers, among others.

An important finding was the detection of *Aspergillus fumigatus* and *A. niger* in the environment, as they are considered significant opportunistic pathogens in immunocompromised patients, and can cause infections known as aspergillosis. This aligns with the most relevant *Aspergillus* species reported in hospital environments, such as *A. fumigatus, A. flavus, A. nidulans, A. terreus* and *A. niger* [78–82].

Infections caused by *Aspergillus* spp. vary according to the location and the immunological status of the patient, manifesting as invasive pulmonary aspergillosis, allergic bronchopulmonary aspergillosis, aspergilloma (fungal mass), cutaneous aspergillosis, sinusitis, otitis, hay fever, systemic mycoses and pulmonary problems [49–54]. However, *A. fumigatus* is the species most implicated in infections in individuals with compromised immune systems and is responsible for most diseases caused by airborne fungi [83,84]. The presence of *A. fumigatus* in this study is consistent with previous research, such as that of [85] who monitored the air in a transplant room and observed a high risk of invasive aspergillosis in patients undergoing chemotherapy, with mortality rates reaching up to 100%

Our results also agree with the study of [86] who evaluated the air quality in two hospitals in India and found that the genera *Aspergillus* and *Penicillium* were the most frequent and abundant, especially in intensive care units and at hospital entrances. In our study, *Aspergillus* and *Penicillium* predominated in high-activity areas, such as F1 and F2, the Emergency Unit, and the hospital entrance.

The identification of 15 *Penicillium* species in the Emergency Unit is a significant finding, as the spores of this fungus have a high potential to cause respiratory allergies, especially in immunocompromised patients. The predominance of *Penicillium* in hospital settings has been widely documented. For example, [87] concluded that *Penicillium* was the predominant fungus in five hospitals analyzed, while [73] and [88] also reported its frequent presence in oncology units and critical areas of hospitals, respectively. Furthermore, [89] reviewed the relationship between exposure to indoor fungal allergens and asthma incidence, identifying an association between asthma severity and exposure to *Penicillium* species [90]. Species such as *Penicillium brevicompactum, P. chrysogenum, P. citrinum, P. crustosum* and *P. oxalicum* are known for their allergic potential. *Penicillium* spores are common indoors and outdoors, acting as allergens that can cause allergic reactions and mycotoxicosis. Some strains, such as *P. purpurogenum* (isolated in this study), are opportunistic pathogens that can affect human health, especially in poorly ventilated or high-humidity environments, where the risk of infection or allergy increases.

*Cladosporium* was another of the most common airborne genera inside and outside the hospital. However, the highest abundance percentages were found on floors 1 and 2 of the hospital, probably due to the air currents that run on both floors from one end of the corridors to the other, as doors are constantly opened. Aeroparticles are carried between the outside and inside of the hospital. *Cladosporium* spores have been widely reported as typical in atmospheric air and indoors, especially in hospitals [91,92]. Several studies have confirmed its presence as one of the most frequent and abundant genera in air conditioning systems, particularly in intensive care units and operating rooms [93,94]. Although *Cladosporium* is a rare human pathogen, it can cause skin and lung infections and phaeohyphomycosis in susceptible patients [95–97]. Furthermore, its spores are allergenic and associated with asthma exacerbation, especially in children [98]. *C. cladosporioides* and *C. herbarium* are reported to be the most relevant allergens, with *C. herbarium* producing the highest number of identified allergens (WHO/IUIS). These allergens show cross-reactivity with those of other fungi, such as *Alternaria* and *Aspergillus* [99,100].

### Proposed strategies for fungal control in indoor environments

Although the primary objective of our study was to characterize the fungal community composition within the hospital environment, the insights gained from our findings also lay the groundwork for proposing potential control strategies. Based on the identified diversity and relative abundance of fungal organisms, we suggest an integrated environmental management approach. This strategy could include:

- Enhanced Ventilation and Air Filtration: Implementing improved HVAC systems equipped with HEPA filters to reduce airborne fungal spores.
- Routine and Targeted Cleaning Protocols: Establishing rigorous cleaning schedules using antifungal agents in critical areas, particularly in high-risk zones.
- Humidity and Moisture Control: Monitoring and managing indoor humidity levels to inhibit fungal growth.
- Environmental Surveillance: Continuously monitoring fungal populations to quickly detect and mitigate any potential outbreaks.

These measures, while not the central aim of our current study, could significantly contribute to maintaining a safer hospital environment and reducing potential health risks associated with airborne fungal pathogens.

## 5. Conclusions

This study identified the main groups, families, genera, and fungi species present inside and outside the hospital. The predominant fungi in the hospital environment included *Aspergillus, Penicillium, Cladosporium,* and *Alternaria*. High-activity areas, such as floors one and two, the Emergency Unit and the hospital’s exterior, presented the greatest diversity of fungi. In particular, *Aspergillus fumigatus* and *A. niger*, high-risk opportunistic pathogens, posing a potential threat to immunosuppressed patients due to their role in invasive aspergillosis. The hospital’s environmental conditions, such as temperature, humidity, dust and suspended particles, played a fundamental role in the dispersion of fungal spores. In addition, activities such as construction and ventilation significantly contributed to their spread. The results of this study will guide future research assessing the relationship between potentially pathogenic fungi and patient well-being, improving hospital air quality. They also underline the need to implement epidemiological surveillance programs to monitor hospital air quality regularly. This will allow for the early identification of pathogenic or allergenic fungi and the optimization of health programs, especially for protecting immunosuppressed patients.

In summary, these findings reinforce the importance of taking proactive measures to effectively manage the presence of fungi in hospital environments and improve patient health. As well as allowing us to propose strategies to control fungi in the air of hospital environments.

## Supporting information

S1 Fig The KRONA graph shows all phyla and genera recorded with both samplers in each sampled area, see Supplementary Materials. (HTML)

S1 Table Alignments with higher scores represent the most likely taxonomic assignments. (XLSX)
