# Supplementary material for: Genetic sequencing of the airborne fungal spectrum and air quality at a public hospital in Mexico City
Source: PLOS Glob Public Health. 2025 Jun 24;5(6):e0004784. doi: 10.1371/journal.pgph.0004784 (PMC12186974; doi:10.1371/journal.pgph.0004784)
Supplement: S1 Fig — (HTML) [file pgph.0004784.s001.html]

Javascript must be enabled to view this page.

magnitude
magnitudeUnassigned

AVPSEUD1\_ITS
AVPSEUD2\_ITS
AVPSF1D1\_ITS
AVPSF1D2\_ITS
AVPSF1D3\_ITS
AVPSF2D1\_ITS
AVPSF2D2\_ITS
AVPSF2D3\_ITS
AVPSOHD1\_ITS
AVPSOHD2\_ITS
AVPSOHD3\_ITS
CuSEUD1\_ITS
CuSEUD2\_ITS
CuSF1D1\_ITS
CuSF1D2\_ITS
CuSF1D3\_ITS
CuSF2D1\_ITS
CuSF2D2\_ITS
CuSF2D3\_ITS
CuSOHD1\_ITS
CuSOHD2\_ITS
CuSOHD3\_ITS

15688121773890158083214849301658867168351717128071561618157733015431031389196280170332169614620991541746156930644408541565235146084513501581803313686409

15688121773890158083214849301658867168351717128071561618157733015431031389196280170332169614620991541746156930644408541565235146084513501581803313686409

18126416325212

124181

124181

124181

124181

18241637211

18241637211

18241637211

18241637211

2205736309175824266221673271736326586348418126033153146200206479236993743980937312842984337341134104370959320115149084

3859201561212667054022374252753934512136128462164285927152826661528703011

3828871541211765943112373152253083292331181425285645910813711318404

3828871541211765943112373152253083292331181425285645910813711318404

3828871541211765943112373152253083292331181425285645910813711318404

3011111872184912731011953064598587270

3011111872184912731011953064598587270

1111294163178832

3011118611720857981951284598504268

3329191252753907364845563822161143916469652337

3329191252753907364845563822161143916469652337

1171911634203615240728159351

1

19179

3328124105389645378213562154611989188061986

55679391

55679391

55679391

55679391

2146912612105320667890109162030235332196330991849984606126710107690205382184105796047407245973205528121

4127269562873539658730571325106829561554585

4127269562873539658730571325106829561554585

1278421170414629763636811364484120

1278421170414629763636811364484120

121466140359304777367200802293

359451042816195444913922683

185271

10463136169

215637777994973

215637777994973

215437487934973

22960

214421258310312046787295162023234426184330843014246054025125488360113209232143110120831994413198

214421258310312046787295162023234426184330843014246054025125488244113209232143085114981994313197

33254706

756420971012231747293109487111463961103126901636387708471495317962516188

13878104861021192475559116201620511617528213186820644299498256354744391474324838319136927003

21

16

16

1162558511

68205851

4851

71515101075854117492354271847035390294727531849177206381551212367

38

38

399474284105544221947725165352386157537392671394941233142

1125513497217833101380102415913131005429

91111015110629932

26624321171515115327141611562446551275

211303310486264134254515101423572204013391198

11117897683516411330162111321525112621434

1231611231167346523124

4118246104519202178402234479285269100

11105271531124

41823610451919165124923347928426796

21115611086481907627613008244425058341456

11120382751

112941050248

211561107518680252569181244422573331456

2140

2140

24112114897111650821791328974814462974175

24112114897111650821791328974814462974175

15672372642910326

156723723419187

308519

10

254189621812084141488580112985

108411217

254189610972083140271580112985

1131232130526612093279945321744722106772483

1131232130526612093279945321744722106772483

201078862118272424518101342543568488708362485135415317749481968

1563901157873137

1563901157873137

20107886218272424518100779542668487708362485035358317740751831

424

354214913678618932992914349531225924282797

1183561773378124106

2237113116982

1710247424271585619459050784551635883229303238731733600846

32124581121113391176447436596601789146825681204

32124581121113391176447436596601789146825681204

2120605923131585672601643135050522711020

21206059234798450188164313155052271964

836117723556

13963323548619972621212

13963323548619972621212

11366712356151411461102176

11366412140151411461102176

3216

2158829320729459319611746

1148715612729378496041

110113780812711705

18822760521842813591208738018563052009029312025518537556283657755297787479341313531101075079328247276566111379

10917269118414947585344949492802207172842456083361735559943224

554227143928333922243527225956471061380353363517731161346

191

1123969553185375077960140545841186196417

2112211235849277583698337996494989942661765449582

111121535227883773270272536750117451270203

232513301615888791271922844963370124811010144

40878057991218

40878057991218

541342141566424612251421647117271781017829086217828781860

541342141566424582251421641117271781017729030217828781859

361561

1113251361521117553

1113251361521117553

1113251361521117553

71514211617167513647397443902555416110901923332450816370395486

7682341313655220579068342405383187101730326440680560074803

7682341313655220579068342405383187101730326440680560074803

9582330961043965747851509114168352531193981511

848212348468356133861202114140050141192713389

11274857569613993072832391268122

11874192609164150

11874192609164150

23104

23104

60852551314

60852551304

1

114951912266715114

114951912266715114

19

19

19

3305138920632123116592503125674118604134221383574911416275427123375555112

1111126761577518928938854266

1657369311011117110

111121912827927827144256

222

222

5024

5024

3295035719632021111232433095546718327231025355104422715388926417374602940

3295035719632021111232433085546718327230856355104415715386526416374602928

11697024112

155691962492482

155691962492482

49211821360241159

1157

49211821244541159

2111534758571812270728144878714770187747

2111534758571812270728144878714770187747

1616522815

1616522815

1616522815

111642551553158290294841468247209683111378313781874112225111793721205

111642551453158290294839466697209581101378293781569321223631775121046

128241

111642551453158290294838466697209381093378293781369317223631775121045

1215512009233824148186159

1215512009233824148186159

1967

1967

4593412269447443280472721225260455654208470137374241575270927583592430907174511605133268

292322117632252782754600498545187247661276931561226

111155617648119744363032221833062

128831110614334213245

1

445

772919422

185015622586186653

2811116011915249523065198766389284723492839838

2

1186

22

113521735450297126155114664156

124274623104

11221

21111018959011737

52416538931101133

521389211111

221139

51132

15906244843082363111686906115854295271366076992164372318504777633699614918554

1490618484298216391574841112402284231069748863157468246804365428338558697183

1612211206534521104296328129690471704122536156221371

11

11

11621216320410169697886112782798389743624231382510

1119521428237212918668

11611216320410169696776612568798361506411321296442

18233112232518302682218318611204925080158226791

18233112232518302682218318611204925080158226791

1142141334278514268506307798413171751973807578125

1411413242777619324730087222704642207455119

11188075259699834294947331601236

13

13

1061681375112257011888

4

1061681371112257011888

196426190962085174478915159612725918821811421925070647

311

111128810583496867290745768211893551939186

1844236184619911114776249871510614721462715333316201

112176812641694638511098108814252

111

11353110048020638749518

1223421421

1223421421

81021115645822533901045210831842115021378229132361007

2721112290351192719339042829524828010362360626

6314416787414638519178913626130981255876381

1710372815372013858651193327935465313103995188151423099557168692924811087

114171133941734259201766136

113420551251430224194552361482417230

821102

46111294136824529308310150708815303490107031771743

1310302714372038453610357373555616869120011545118209278615316248868978

12643471193112122

12643471193112122

117

117

132113311518189495728173429213172121461423662127109

1

1163966221686

32123175769435872443303221094157040819442042103

1147495731307397114378470878846

36

28187527536

2

14463511187601535322501865718791913020771729564520657274

14463511187601535322501865718791913020771729564520657274

13123111082834217561405869163151618573501159

22064567118910866441915431441759

873171212411545565134118

13121113206356731910115122073148199982

131384

131384

12037733413118525701635

12037733413118525701635

5

5

112768991873648

1261873648

1261873648

1276873

1276873

7910111251804258139242804304779121883292636256416344633234

547923159743625417298269711960187920082906166021681924

547923159743625417298269711960187920082906166021681924

49534323250111144631161184

49534323250111144631161184

253212201816541698215844545139177051344011341126

71484322197226

25321220181654162681584453673595685434401134900

11

11

5421122904324623962044441193473276758803

5421122904324623962044441193473276758803

11091275811111

5321122904324623961935441180722465758692

1001848422982981087776528817578654456619087273032192551989952996425408921361873397101633326911033941

1728141016639372943

1728141016639372943

136

136

114471122368

114471122368

722581185823548115452681742693245231189602579148655157405239818344261936952102981087224438718333

19108427138882976132215412099136469265543242355131145381241627078534016121939248

5314971586847398247526217294717448190904661101221011249821846871988806953313951374706321949085

3675124454568772449690682345

6723226023

35124454338552389690659345

239524711111212483912808255249627142572564152731304334503030

393

239524711111212483912808255249627142572564148801304334503030

22361113141485139304608335277913390345105216121553465951

473158151

22361113141485139304608335273183390345105058121553464900

24131549713123891832613292406980740944

24131549713123891832613292406980740944

113803180519939116382925

1380332919939115382815

1147610110

19158612749601819181081342681869102277872689929941132765332659005328473149358801

211210282782649867719271133

111231

11121

21

111575917295952141493737988251926726916811530889701280230488584665673868

2752531112437221119513146169520332464844497031551395

317720101315516365377191426110346147873851215575321681697546483526

6247404593845058569723

1

1332129401661352967151631207046785515

42354501

174

215231263005675626505647113654804875157469622

1152392988660824135418113644174207149468622

1117171482376216152871

16721401

12112111523816017062886427510465743149887

12112111523816017062886427510465743149887

23

23

23953966210410789615245591636281144020442305617466126385410482

1375622308313

1375622308313

23953966210410789615244591636281144016262304995460325555409431

23953966210410789615244591636281144016262304995460325555409431

432848

432848

1111812356292040399820361145833611628796773031290495

1111812343692040374220088129933611628773843030275195

54

13

74

101612328853653238153751012131446636232913241543

11903114881043346140128

1512224631

26132

1015693

21

941193

112388473738845733442125506113214

2021111282204

126111012771

1261

11012771

25527214910161152

25527214910161152

33259119219492037471214183154672051547329089209091701895961490991214452

33259119219492037471214183154672051547329089209091701895961490991214452

33259119219492037471214183154672051547329089209091701895961490991214452

413224604128555661550719991806952754401922466401

413224604128555661550719991806952754401922466401

413224604128555661550719991806952754401922466401

413224604128555661550719991806952754401922466401

114690266429281508141951143250332821576603

114690266429281508141951143250332821576603

114690266429281508141951143250332821576603

11

33211996

2874216212871838102211932247215568

91144710

114341

111312267662241569821016539135325

81218164295382034826414142809166841536226767504090873048370721701357

81217163995382034826404138807563731409624476551387002492307820071171

81217163995382034826404138807563731409624476551387002492307820071171

11161119413116

56161638953820345429293003199618926551368418476661751324

16114426404084514622091171651310991202742243

126542347327167013847

3

3

2

1

1

1

1

1311113

1311113

1311113

13141629412662289527387556518163183

342808952435621623112

20

342808952435621423112

111614126622033155435616071

158

11163111822031523215265

3223

2031

8126301186

13121016024122545932821172751634091013

13121016024122442491791110243831471013

13121016024122442491791110243831471013

13121016024122442491791110243831471013

1182791219

1182791219

1182791219

10354666243

10354666243

10354666243

162

162

162

148

148

148

148

1311224312657546832811058126171856148110571346865

1311224312657546832811058126171856148110571346865

1311224312657546832811058126171856148110571346865

1311224312657546832811058126171856148110571346865

116534374631981726187930

116534374631981726187930

116534374631981726187930

116534374631981726187930

128211

128211

128211

10

12821

12021852223281354412200

20034422813562198

20034422813562198

20034422813562198

111643181472

18146

18146

11164312

11164312

121

121

121

175367

175367

175367

154631017372691514301145998316362451682817153877711453321576851142975512630821265901319320663547603780758294315643511307563240829789051481246537052

11146120922802659

11146120922802659

11146120922802659

11146120922802659

11144108121444123840168

11144108121444123840168

11144108121444123840168

238160

148239

114293143765626

327262

965010

1950208217561542412089153167121661571514523448561171593325879176607172404043898804136651586759843055181417625649446967462921

116132545482258

1612554548236

1612554548236

17222

17222

4141113241321111381543355833761181624012366652844185825874

1125211155031943243955589310170513104211

251149945218332558561145139579

112150914221663430456852

1916123823822341203162796431

191618822341203152896431

12202399

1213496952181313522178223016

1213496952181313522178223016

223124321018786832922269794916040251235038944547416

1

19602285882325476

22324321018777232922246994916040192411838934493340

2257

2257

2253

4

24643125130302311375027492109820287451576133988903134438550633737301675

5614156146523618243381152480126973531218136411371214613

5614156146523618243381152480126973531218136411371214613

1101214721

1101214721

111111121274127482204471143438716108343302169108

4

111111121274127482204471143438716108343302169104

1857169113151458648961550232496790253116622117684327512345953

8616142

1211223103230173282252821044

2535216971514

185614911314133864894149393131670824989212028680324642227947

2026184219264417684018205132071672923055197192479015601728747744551237833450325422

172615361726421750391517311176160363303699139425581390752206572336186502515722119

172615361726421750391517311176160363303699139425581390752206572336186502515722119

194

194

6

6

21221882275195412

21221882275195412

315218131120316921174577622313169366681193010512993283303

1

31521813120256060156362195859036599180010512690363004

6444952846232154128

1

134351479914811290

7417311150

2

4079239

70

15

11

141273448365553708441363437715211431299130264131579010808263931142681196383286834117714673408145017263535221560177031

811656521261

52

8161261

165

112264622206055

11226492203765

53229

13787344436546370823136336762521134129792956363157459255256534139154192340259821113723572243842946251630213133149352

831411219213861

12224569079254933411248365238448767

1

13787344436538370820136336761521130129792956353156339233254078139063191548259180113389372080342581251391212685148585

321416529514387536421048937180051283586202885570393725838

1277614211869

26111682149

1141294160

211

1301442205

11117610593671646955

12

10381

4131537314387016373629102921220048455536145220579

129440317636169187

1112251999267461245132305817443687

13

128555378648102

62212

2355216324267837502479310687192706827591627611334481822

22167358193586202189140103175

21117131087644085114560218141017646

212232725741272925474165

28

12111131034844111714529144653452142030

133631322185163

1121211114411881019157613218649563

11112846231168702328618552621093

112943329994618614397

1

181508678572103390

190

119

4801

17314

17314

51224213

141421

4813

1081419105

1081419105

105

1081419

490132086524910411881404839594479147744497457249827616631935745666583758720503195297108152301224983180760257646

211133682134210931277161316

1113314213411193316

2541109227768

324291201026

323291201026

1

71781138189781955748645100754185645300217215171831450040750141901837915216

71781138189781955748644100023185645300216858171831450040750141901800215191

173035628225

194

1

1

31441631481158676163736222589

11137982

313416012583561432664

12146

118311359943

11

11

12013113129116772913633000823331713754604980

111612

14226191

21309

135589

261

12031212911647231343241532333583687482872

33

11328

28

2

15

11111696542212819266754222915

1

1

112166233554348228

11167654551342413193115

35

1

88

88

12012966412918811494631566264673441364915895012185118383032719141828708380997451140831170144

12121376972458

9112965011214011415623462393653262098820473957966349313135339920411072312812928963343

111

11911

1

3111

331

1

1

7211161279

111

1

22126954998756551084411633621973311

112

26415467811219253611513796329671295513720998451981833925967421510467102608

2116182102

1633112134622

435111113237

1114301313243105330237239257

1114301313243105330237239257

111111511835881031012628442673

111111511835881031012628442673

161414410921

13118461

33551032

40

1

6171073042635835163183814207612004276378872274476248101558352

11175

11

1191

12

161112401641

21169143865176555522012516965871816758352735

2522393

3314111156207372098711517220547503773531

851106360

34712916

137

12238263908

21

1

11171612272

222606574

46527221431913074

16441117112841

412462

332121103582082112845967488616917613213465

16

11242471125001249

1314151480213899830637758621481621239

1371811618473049816269

8191214120791531

8191214120791268

263

18423255138

138

184

23255

1502339186751601422458471444

1502339186751601422458471444

119740311113031

119740311113031

3335410

3335410

1121197313268225148244340604734601405

1121196312268125148244240604034551402

11221

11532

15171

15171

339233524745398514923189284710184447636324911658157277191451208840395819012328137415884277739670

435

221423521029139134334802616

39433

336233524722397034893189277910084447635724898153687264180051193236152770211887133215548185136640

261261334462614156133048333334937

1642111

1111113337

1741139

1219

5

5

983515485121724039

1

982515485121724039

2462042343

2462042343

8278911007136181167231189202516801426971187759116510653511

3891110

175161

3114

125

8272710967036455211440811368801737941502693

41368

121211127851617157826156452615115363

5110014569142

2173

1

152219196299

1651111920111779621113541014009201010077

1651111920111779621113541014009201010077

533111212249153731859522971065689964

533111212249153731859522971065689964

2112394099260210242037212054226273

68

68

423199221257225144

423199221257225144

21123940992111102445197971129

1211596219113211

11250269010134214678

11118423118129

1

1

1

1390273152777095211310232821479496168121992187669144214669451341087628919188081632122602777277034712710621790146029212074788750617451

1543406139032055

1543406139032055

24910

1541113896145

9051259

1113526111

1113526111

1113526111

111

111

111

171303730300343651311141235316618716267243741413771805117552416116797143464274

334242111492155164326774185676681810572316268

8

3242421114116419569018567521188922316268

1215513184147165

4612715133736524501830294125424129163159915228

4612715133736524501830293124424129163159915128

111

21113531310124901419211147786111520710369758

5372

8

15

21135031244414151781631261550

1

27197

131100334301287858431861021978

4123732725232126594785585202013775083658592517681574871542458

27461158531254

4123732725232126594510985202005963503605592417653574869002458

7828

41141610115347272091865334340642534525337565624835746271462

11341111263134619

18632743814713394

4783321121005861176710592889362325644868159884252

155342133234433242553201075990122453556504136041184

1211190101310141659119718141333

4523456372521618133134691702442467847968

4523456372521618133134691702442467847968

130145

41

37135454

113152156309871008131447

4422426362160374341692368041891

6443

2

111214295162687016645973411891786937927153

58

58

1112751623866124933213758156089266131

112691616804122828710649153689247117

176220303118196

108

1511568

5133199

133199

5

11141534154213184811121

11141534154213184811121

414784281673511

25

423

213511

41455167

139025215276339520731023248147918916811809215046914251466927134107162889814979937462429734607919499638605656023404810050287090312961

17169291412624031285248263285174749121621631389701212099143387062221

5434623111121144513154853925361483724676460

92298451691038813261331723

314317129211223116319254283633797036158504452615136139575161

13901881527442951986102304614788871681053918335690135146687413407706285881495785498241748427211797063848865464318109786178365112708

120816514256257549278005111323325111323957708154004766419112928765836581257343983189682284201137337588140674224534565873288810053

1422

118639

18202210181719705922253515556256781434125415008880268347894449302384415015206614301659726260724790933952203507631994

4722586140874112385382553499911951404717139120819032

452258613984412353825526219660140471712412081184

2131215878535157228

122350336214

122350336214

2235099

1336115

96612342419

96612342419

96612342419

96612342419

38223523806261924165221161943395854248620064355178646368334356234533361026

38223523806261924165221161943395854248620064355178646368334356234533361026

31812521222011321321283233339

214308

2185755452314021

1667

11

290231

11152057742292

1312165877523312734388351731791316972111211302002521818471

125881201112211412

1

1211143655311212332762333641655914298587629383189151037

1121111111633114176796448113191

122126720220517203068993228

111115463861034551223978511783

4

3

3

111136734208812477285356150

1117

11112673420881247728535633

11111228861283928271119321125

15395212

111111278512239281641421123

5132232

18814237801110117165143010583264911290904883522

11

18814237801110117165143010583264911290904873512

4417714211099981192101100236459333143456073724723

1135141269573096812

53221149313191261749032802312

112

1221254112254114232337

2225121114540253210815581312061431104

22835221194754432463187426810110

5

21111881143402763627884131482456625508244

11151032221

1621121

111181143352660625837101472454417397241

452061111

40214121402602

260

40214121402

111264312021

111264312021

13367519118538944844160774476146

13367519118538944844160774476146

73

73

3207909532

3207909532

24

24

1162191300348446545045663

141190300126534544262

12112484215141

18510183

101

18583

2111815100944499487701122356294321

2111815100944499487701122356294321

2111815100944499487701122356294321

21832153252

6

11136117

2222

31546329638

21181100942545087551113128021

206851064211383047354271061420541922721107262263387331136547152021059053200142598

18

18

18

74912948164547967605888592084611116385021368162634522172

1

1

22221232212704338661123912448330

1422

2222123221266338661123910248330

3

54710736132547946335884558283951115182631244162233692142

11121121

3158

11101134

211

211416238466141

111

3361072591537946326882547083551114382601041162032062141

1127493327301341293844051292881761414927253559368527126027124651035462775038496

1

1

12

12

11112335721394521955

11112335721394521955

11273723342592332157854340536350

1273123282238225417444321526322

1

3134616633812119

135810379

634720

634720

11010322327792934141280363883375571103

156112045

16781224531

7627923949920160101146619

18

2

1109322321413551031518121245265

31

31

2251111322911270762240121653615991565215824631488

3113182131320

1211111279811119258281231185826378

1

1131

2271633291154

253

1111226104534791201209331471348214920421090

185726100831111538765

849011411418645

572282922011

1

5

2711

75981284261039292341661179081524697012473013683041204699641969862458130545

1

5487320317429221040664763491533359199647329146916156776640101176126252

11248113

12641

11133123348911471371251

6011

1107861952966950752515838448063914828638253832959122023964

119

10414046779187

52134322612434230

3171111

171111

3

111

4

111

18211051

111328111228281956108

21140

135491141102

1116521

1116521

1116521

12153523512220013411663

12153523512220013411663

11

12152423512220013411663

11121190436264721384376402513697336331926

11121190436264721384376402513697336331926

161323410823136922

135

111111903361734010433762727316331924

141230194551519309914859547415220811474572

26

26

26

667714371340

37257

3787

170

1

1

66771483

424

66371279

2303421169911

2303421169911

52

2303311

33711664

1412301945103865711485732381131425246486

1412301945103865711485732381131425246486

224

1412301945101865711485732361131421246486

5

5

5

21112

21112

21

3

109

177115192609

177115192609

177115182609

1

1

1

1

433975

433975

433975

221

221

221

54137

54137

54137

15402612495236893514352112343931764998874364067910125307795965429702037748361911559163149911967010666511641

111211124331076718115641778417603191730

111211124331076718115641778417603191730

111211124331076718115641778417603191730

21131354914072396817015257318

21131354914072396817015257318

21131354914072396817015257318

2331412087172513546852546446216911521001324814

1221002716164350874571163415416183686806

121164149912442154118824504

1

22

67

58438114554317597

224022759699608918412

4

129

23642

11312206001870381683481262853463325688

11312206001870381683481262853463325688

3131231353

3131231353

33

13123135

6472

6472

6472

143113101373582145767156121547412813582478495678

6351141292311

15111

63511402872

1137566649653633851125561623256638295150

1137566649653633851125561623256638295150

222983

222983

2

2

13213708724601876120873819341023340136318

12124896496495428509628328339109011

525111613125

12121861713138062216130567012737

1347556165061615707614976407

1720416047504231

21

347301302132251073573

1989186

552

2011

67

11

11

10381

10381

1

1

47752442231133712

1257243117056

1257243117056

35181161128112

6

349011601280

2

1512

411

1

183528

1578

1578

2652

2652

15284722836947426452443313397336495687307861131772848216044111056966310222893640135843270770

315

315

2211774817127462394213001284

1135

292

21774828974611300

1423

47

11821236

1

11

2

235

15284722836747226452443313397326495587307861131772073014270103546711310122883219132841934770

24233706251111121

15282322836546926422443313396626494987282861131772072914269103546710310122873219132831932769

162511152

1

111

162551

11142503218119007510669424356303

11142503218119007510669424356303

11142503218119007510669424356303

122513981718551679260316

122513981718551679260316

122513981718551679260316

34413611305711110793112751978525691113955250368920

34413611305711110793112751978525691113955250368920

1

11

34413611305711110793012751978525691113955249368820

12

12

12

53171894994886244

53171894994886244

1871848

53112113

620686244

1740643303074754211624459187928353144656710962102077347891672727375709892638

21251812326128354

21251812326128354

11378302530080421333146351

11378302530080421333146351

1111

1111

31291314443152580148502653586758151275515725922913182

12

31281314443152578148502653586758141275515725922913182

1

12171115576211

12171115576211

21111311265311119722062863856321815307124379298

11511930173514451854627011265131

858

14349028

1

6

11122113221448338537701671048545

174722126982214112431177194

2

1112871

145239511165201275811477382771593734282681234242060135303233

11113

419313223

311923382536278247

145209511165201275311164382761591700122675833922032334896233

1160

22233211319220410812456547774276615772453929

114181053205810022

2223221831784312947554815742452891

4882124433226271136

173342170

1342170

73

213315414557286463107819

29443249821419

1160

1101

32101100463100

124714763

164

1

1

4388842943118175291487248072898171994602103265137223880553

195251146661

211642122122224313280110

31732112145291406947462895156994372099216733053532281

104

1176085116159

135144162

38712922111

2137

4

31552

11113207638025114374338118302

11132076982374333718302

13801531244

12268529427054304204362188319083272720529

411927

411927

12268529387054304204361188218993272693529

692

1511

23

227223

1234929381821786111291257119598

33511

8932

215222523354359105952610370210

141821651040246513119981345801231990782

1354110190354

1354110190354

324

324

4181808104024637911998134390877990782

41322557191199863382710

483101

1696875

1

5892353701285299071

1120604881585517372599521188232066796378351588914325136041080928820524015694153114488

134313030371552386150652156253270018281020

14536

1305002351101939343259498131

134220841552151139011694634411330889

33

26

11414431702412864920611733923364277943

123322

13064

114443170128206492043173322336277241

11

11741

422512742908165472166196854522312227514

24718971

841

1

28

1

51

5

238

3

1118121316541835096814517414

56849

41515216951230372315331

111754801227466722995011634320664292279780378591701261592690725071192448622981

113731986102877

12232915383972911

11

9838

1113547696726560994891529320654021157924842891602926176681319941002723572325

124166229

1342541

1488

64

1944174243116

22514940101

22110011321596550891464238236362197929942212

11154511150125

111116414739258821844006211056758489

1155293291422

10364

1

2223136063162551327

1498123421

2111130361872914181129354174622

1303621353693

211129123850174322

17879

187

310

1

1

331561422815

331561422815

54423470241181451364222624911012611767

54423470241131452412252496212611066

5112410148701

1935415

1935415

1811512443141519379948219368961532274640396

122015813018773727968159378

14382361

16

211111235111445

17875

1

41123368218292210816094143437191312

3115211352444286925612332688219432524491676580

3115211352444286925612332688219432524491676580

3115211352444286925612332688219432524491676580

3115211352444286925612332688219432524491676580

384171013522

384171013522

384171013522

384171013522

44531264773681455373398409700131112930814333046057523163811574226342921951271

44131164770679448366394409677130112925773918954581131278506361509371803138

11111321741

11111321741

11111321741

441311647696794483663944096771301129257739089545811316550636129237180397

111133743021104922721266364449

111133743021104922721266364449

917272

2175

71797

440310647686794483663944096771301129157735715515631164836491148748

440310647686794483663944096771301129157735715515631164836491148748

41327742315442215599410360651061125255148133

43267323153718631335316547998962462184

42266322153718331134916143988922451581

12227152524828114733

12352201451551592977436897552371178

3231211139512311

1113244414163

1113244414163

95111643217

95111643217

95111643217

111126242865679144229712732

121228431722128331

121228431722128331

11124725360111853630

11121151121

1246525961842429

52111611181

52111611181
